# Occludin is a target of Src kinase and promotes lipid secretion by binding to BTN1a1 and XOR

**DOI:** 10.1371/journal.pbio.3001518

**Published:** 2022-01-18

**Authors:** Yunzhe Lu, Tao Zhou, Chongshen Xu, Rui Wang, Deyi Feng, Jiyong Li, Xu Wang, Yu Kong, Guohong Hu, Xiangyin Kong, Pengfei Lu

**Affiliations:** 1 School of Life Science and Technology, ShanghaiTech University, Shanghai, China; 2 University of Chinese Academy of Sciences, Beijing, China; 3 Molecular Imaging Core Facility, School of Life Science and Technology, ShanghaiTech University, Shanghai, China; 4 Institute of Neuroscience, CAS Center for Excellence in Brain Science and Intelligence Technology, Chinese Academy of Sciences, Shanghai, China; 5 CAS Key Laboratory of Tissue Microenvironment and Tumor, Shanghai Institute of Nutrition and Health, University of Chinese Academy of Sciences, Chinese Academy of Sciences, Shanghai, China; Ecole polytechnique federale de Lausanne Faculte des sciences de la vie, SWITZERLAND

## Abstract

Lipid droplets (LDs) have increasingly been recognized as an essential organelle for eukaryotes. Although the biochemistry of lipid synthesis and degradation is well characterized, the regulation of LD dynamics, including its formation, maintenance, and secretion, is poorly understood. Here, we report that mice lacking *Occludin* (*Ocln*) show defective lipid metabolism. We show that LDs were larger than normal along its biogenesis and secretion pathway in *Ocln* null mammary cells. This defect in LD size control did not result from abnormal lipid synthesis or degradation; rather, it was because of secretion failure during the lactation stage. We found that OCLN was located on the LD membrane and was bound to essential regulators of lipid secretion, including BTN1a1 and XOR, in a C-terminus–dependent manner. Finally, OCLN was a phosphorylation target of Src kinase, whose loss causes lactation failure. Together, we demonstrate that *Ocln* is a downstream target of Src kinase and promotes LD secretion by binding to BTN1a1 and XOR.

## Abstract 2

Lipid droplets are an essential eukaryotic organelle, but how they are secreted has remained unclear. This study shows that the tight junction protein Occludin is a phosphorylation target of Src kinase; Occludin binds to BTN1A1 and XOR to facilitate lipid droplet secretion in mammary epithelial cells.

## Introduction

Lipid droplets (LDs) are microscale structures that are ubiquitous in almost all eukaryotic cells. They are composed of an inner hydrophobic core of neutral storage fats, the triglycerides (TGs), or cholesterol esters, and an outer layer of protein-coated phospholipid monolayer membrane [1]. An important means that a cell regulates its energy storage is by controlling LD sizes, which can vary from less than tens of nanometers in diameter in most cells to hundreds of micrometers that can fill an entire adipocyte [2]. Although initially considered as passive energy storage depots, LDs have gained increasing recognition as a new organelle essential for various fundamental cellular processes, including lipid trafficking, vesicular transport, and metabolism [1,3].

Contrary to their relatively simple molecular composition, LDs have a complex life cycle comprising formation, growth, and maintenance stages [4]. In certain cell types, including hepatocytes and mammary gland cells, LDs also undergo maturation and secretion stages [5–7]. Briefly, LD formation starts in the endoplasmic reticulum (ER), where the accumulation of TGs in the shape of a lens forms in the cytoplasmic layer of the ER membrane. Through a poorly understood mechanism, these micro-LDs then separate from the ER and enter the growth stage, in which LDs grow [1,4].

An LD may grow via increased lipid synthesis or, to a lesser extent, the fusion between 2 existing LDs [8]. Because there are in the cytoplasm multiple forms of lipases, or TG degradation enzymes, for an LD to grow, it needs to be shielded by coat proteins such as Perilipin-2 (PLIN2, also known as ADRP) from these cytoplasmic lipases [1,9]. Thus, an LD grows if the local synthesis of lipids, catalyzed by enzymes on LD membranes, outpaces degradation. Therefore, an important mechanism of LD size control is by regulating the number of shield proteins such as PLIN2 on the LD surface. At the maintenance stage, lipid synthesis reaches an equilibrium with degradation.

Lipids may be secreted via one of 2 independent pathways. In both hepatocytes and enterocytes, for example, lipids in the form TGs are incorporated into lipoproteins, which then take the conventional protein secretion route and are secreted via a SNARE-dependent membrane fusion event between secretory vesicles and the plasma membrane [10]. In mammary epithelial cells (MECs), however, LDs are secreted via budding, i.e., mature LDs at the apical domain of MECs are enveloped by the plasma membrane, pinch off from MECs through an unknown mechanism, and enter the alveolar lumen. With the addition of lipid bilayer from the plasma membrane, secreted LDs now have 3 layers of phospholipids. They are sometimes called milk fat globules (MFGs) to differentiate them from intracellular LDs [5–7].

As a major secretory organ, the mammary gland is an ideal model for understanding the mechanism that regulates lipid secretion [11–15]. Loss-of-function studies in mice support the involvement of several genes, including *Th-POK*, *Cidea*, *TDP-23*, *Btn1a1*, *Xor*, and *Src* kinase in lipid secretion regulation. Among them, *Cidea* and *TDP-23* regulate lipid secretion via modulating *Btn1a1* and *Xor* mRNA expression, transcriptionally and posttranscriptionally, respectively [16,17]. By contrast, *Th-POK* indirectly affects LD secretion by transcriptionally regulating lipid synthesis [18]. So far, only *Btn1a1* and *Xor* are thought to directly participate in the interactions between LDs and the plasma membrane and the secretion event [19,20]. However, the mechanism by which *Btn1a1* and *Xor* regulate lipid secretion, especially how they facilitate the LD extrusion process and what additional partners they might require, has remained unclear [5–7]. Likewise, how *Src* kinase regulates lipid secretion has also remained elusive at present [21].

As a founding member of tight junctions, *Ocln* was initially considered to be essential for tight junctions’ integrity and function [22,23]. However, recent studies show that *Ocln* regulates an array of developmental processes, including stem cell biology in the developing brain, which are not directly related to tight junctions’ functions [24,25]. Likewise, we recently found that *Ocln* regulates milk protein secretion via SNARE-dependent exocytosis [26]. Here, we describe an additional phenotype in *Ocln* null mammary glands in which LD secretion is defective.

## Results

### Lipid droplets are larger than normal in *Ocln* mutant mammary glands during lactation

**Fig 1:**
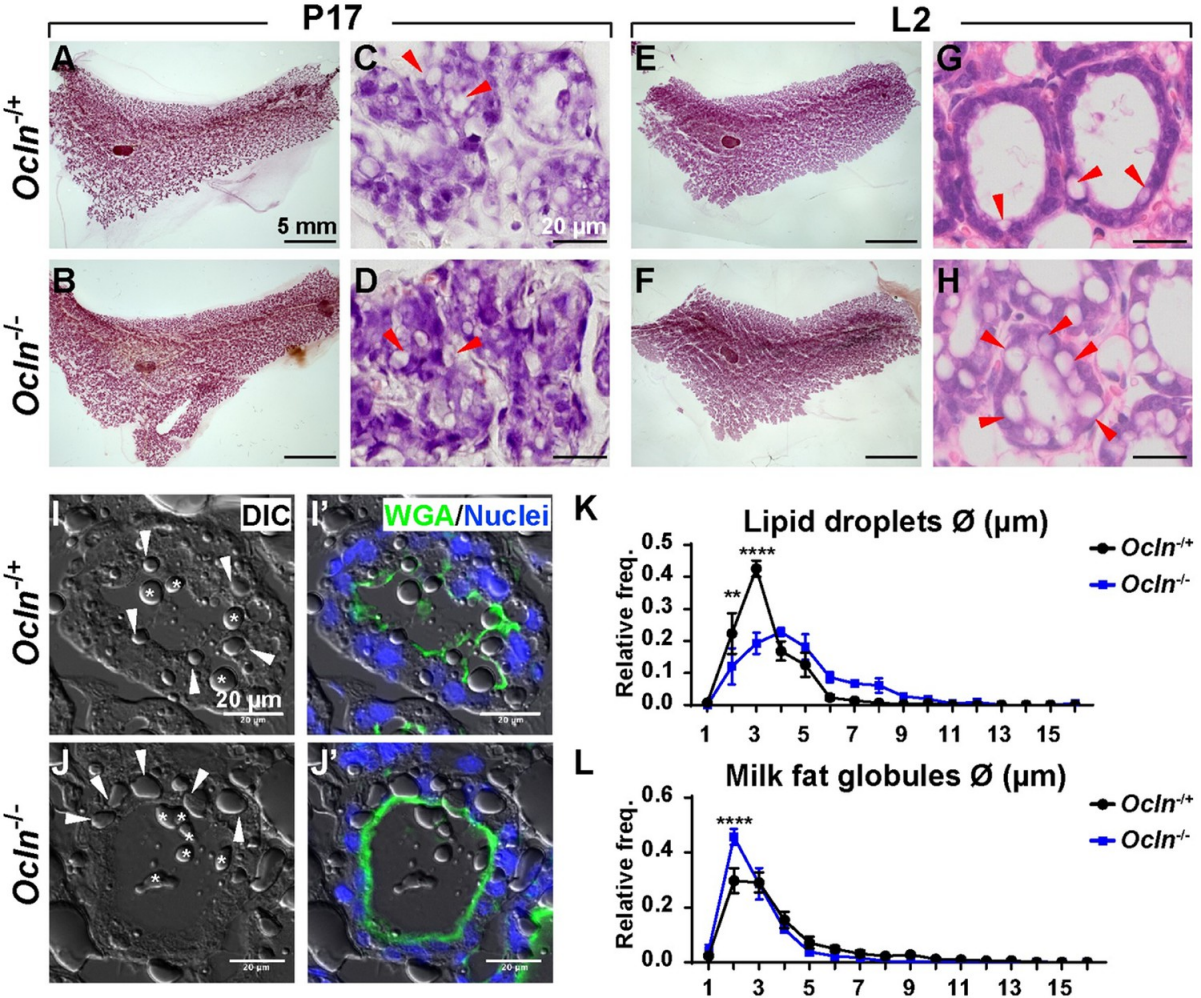
Lipid droplets are larger than normal in *Ocln* mutant mammary glands during lactation. (**A-H**) Epithelial morphology as indicated by whole-mount mammary gland staining using Carmine Red (**A, B, E, F**, scale bars: 5 mm) and H&E staining (**C, D, G, H,** scale bars = 20 μm). Red arrowheads mark LDs. (**I-L**) LDs as detected by DIC microscopy (**I, L**). WGA staining (green) marks the apical surface of the alveoli and is a demarcation of the lumen (**I’, L’**). Asterisks indicate MFGs. White arrowheads denote intracellular LDs. Scale bars: 20 μm. (K, L) Quantitative comparisons of the size frequencies of intracellular LDs (K) and MFGs (L) between *Ocln*^−/+^ and *Ocln*^−/−^ mammary glands. The number of female mice at the P17 stage used were: *Ocln*^−/+^ (*n =* 3) and *Ocln*^−/−^ (*n* = 3). The number of female mice at the L2 stage used were: *Ocln*^−/+^ (*n* = 6) and *Ocln*^−/−^ (*n* = 4). Note that the individual numerical values that underlie the summary data in the current figure are listed in S1 Data file. DIC, differential interference contrast; H&E, hematoxylin and eosin; LD, lipid droplet; MFG, milk fat globule; *Ocln*, *Occludin*; WGA, wheat germ agglutinin.

We used Carmine Red to stain whole-mount mammary glands to examine epithelial morphology during pregnancy and lactation. As expected, we found that the mammary gland was more developed, as evident from more densely populated alveoli, at the lactation day (L) 2 stage (Fig 1E and 1F) than the pregnancy day (P) 17 stage (Fig 1A and 1B). We did not observe any gross morphological differences between control and *Ocln* null glands at these stages (Fig 1A, 1B, 1E and 1F). To examine the histological details of the mammary glands, we next cut paraffin sections and stained them with hematoxylin and eosin (H&E). In the control MECs, we saw a prominent presence of LDs at the P17 stage (Fig 1C) but far fewer LDs were observed at the L2 stage (Fig 1G), presumably because most LDs were secreted into the lumen at the latter stage. Interestingly, while we also observed an abundance of LDs in *Ocln* null alveoli at the P17 stage (Fig 1D), many LDs were still in the mutant MECs at the L2 stage and they were bigger than normal (compare Fig 1H to 1C, 1D and 1G).

To quantify the size differences between LDs from *Ocln* null and control alveoli, we cut frozen sections of the mammary glands at the L2 stage and imaged the samples using differential interference contrast (DIC) microscopy (Fig 1I and 1J). Wheat germ agglutinin (WGA) immunofluorescence, which marks the apical membrane of the mammary epithelium, was performed to differentiate intracellular LDs and luminal MFGs (Fig 1I’ and 1J’). We then measured the sizes of both LDs and MFGs and performed a percentage distribution analysis (Fig 1K and 1L). We found the sizes of both LDs and MFGs varied from approximately 2 μm to 10 μm in diameter. Interestingly, while LDs of 2 μm and 3 μm accounted for a majority (approximately 70%) of the LDs in the control glands, they accounted for only a minority (approximately 30%) of the LDs in the *Ocln* null glands. By contrast, LDs of 4 μm or more in diameter accounted for a majority of the LDs in the *Ocln* null glands while they were in a minority in the control glands (Fig 1K). MFG sizes were more similar than LD sizes between the *Ocln* null and control glands, except for the MFGs with a 2-μm diameter where they accounted for approximately 45% of the MFGs in the control glands, but only approximately 30% in the *Ocln* null glands (Fig 1L).

Together, the above results show that LDs are larger than normal in the *Ocln* null epithelium at the L2 stage.

### Lipid droplets are larger than normal in *Ocln* mutant mammary glands at multiple sites along its biogenesis and secretion pathway

**Fig 2:**
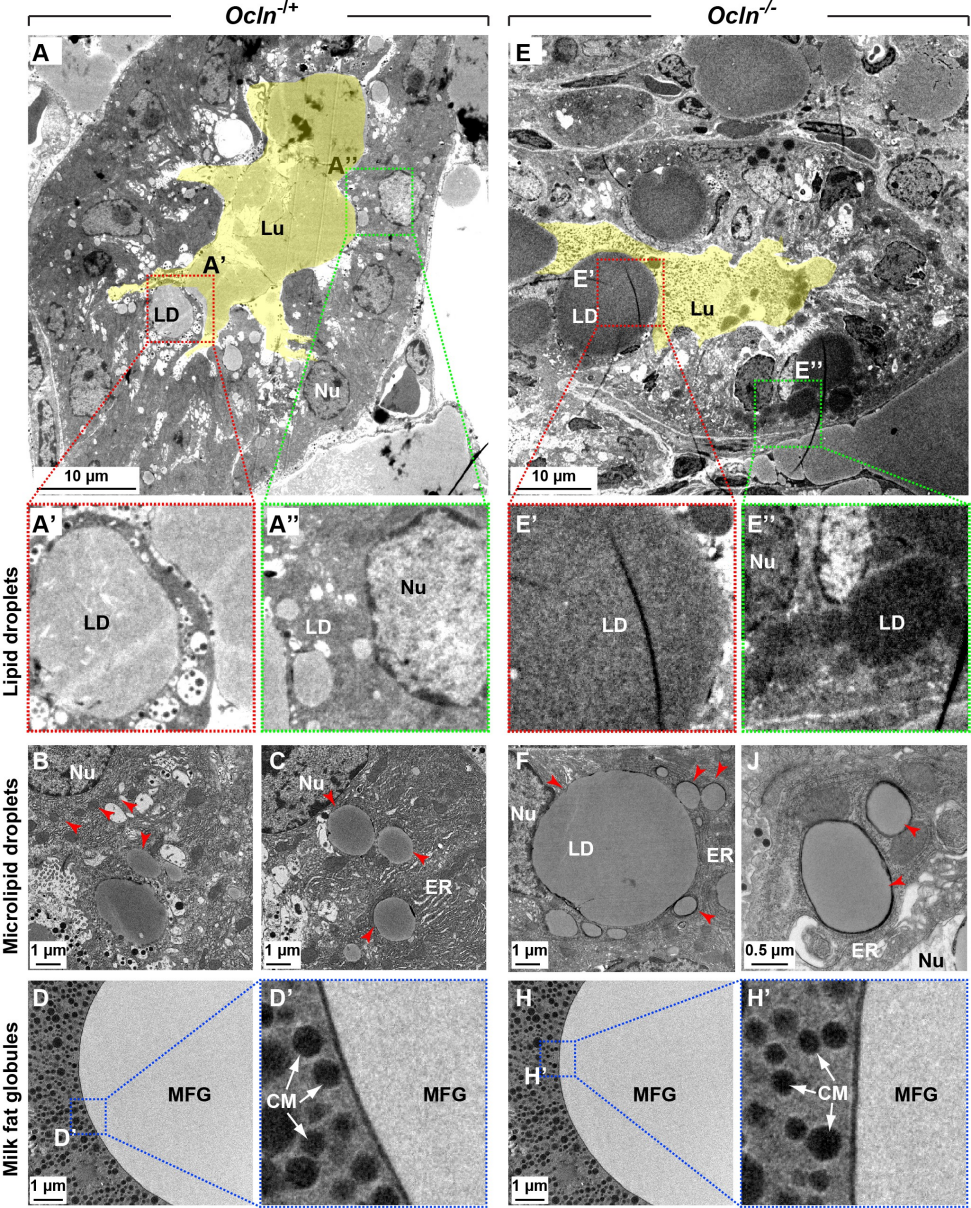
Lipid droplets are larger than normal in *Ocln* mutant mammary glands at multiple sites along its biogenesis and secretion route. (**A-H’**) Micrographs of mammary gland alveoli from *Ocln*^−/+^ (**A-A”**) and *Ocln*^−/−^ (**E–E”**) mice as revealed by TEM at the L2 stage, the lumen is pseudo-color painted in yellow. Areas in red (**A’, E’**) or green square (**A”, E”**) are zoomed in to show mature LDs that are apically located (close to lumen) or microdroplets that are those close to the ER, respectively. Note that both mature LDs (**A’**, **E’**) and micro-LDs (**A”**, **B, C, E”, F, J**) are larger in *Ocln* null cells than those in control cells. Red arrowheads indicate microdroplets close to the ER. (**D**-**D’**) MFGs in control and (**H**-**H’**) null alveolar lumen. Areas in squares of dashed lines are magnified to show that the thickness of the trilayered membrane of MFG is normal in mutant MFGs (**H’**) when compared with control MFGs (**D’**). A total of 141 LDs from *Ocln*^−/+^ alveoli and 211 LDs from *Ocln*^−/−^ alveoli were examined. Scale bars are as indicated. CM, casein micelle (white arrows); ER, endoplasmic reticulum; MFG, milk fat globule; LD, lipid droplet; Lu, lumen; Nu, nucleus; *Ocln*, *Occludin*; TEM, transmission electronic microscopy.

To examine the LD size difference in greater detail, we prepared the samples from the L2 stage and imaged them using transmission electronic microscopy (TEM). Consistent with the above phenotypic descriptions, we found that mature LDs, which were apically located and close to the lumen, were much larger in the *Ocln* null epithelium (Fig 2E and 2E’) than those in control epithelium (Fig 2A and 2A’; S1 Fig). We found that nascent micro-LDs, which were embedded in the ER and closer to the nucleus than mature LDs, were also larger in the *Ocln* null epithelium (Fig 2E”, 2F and 2J) than those in control epithelium (Fig 2A”, 2B and 2C; S1 Fig).

Previous studies show that *Xor* function is essential for LD secretion and MFG membrane integrity. In the absence of *Xor*, MECs fail to secret LDs, and MFG membrane integrity is compromised [20]. Interestingly, we found that MFGs were morphologically similar and had smooth membranes with even thickness in both control and *Ocln* null alveoli. These data thus suggest that MFGs were not defective in the *Ocln* null epithelium.

Together, these data show that LDs are larger than normal in the biogenesis and secretion pathways in *Ocln* null epithelium.

### Normal mRNA expression of genes regulating triacylglycerol synthesis and degradation in *Ocln* null cells

**Fig 3:**
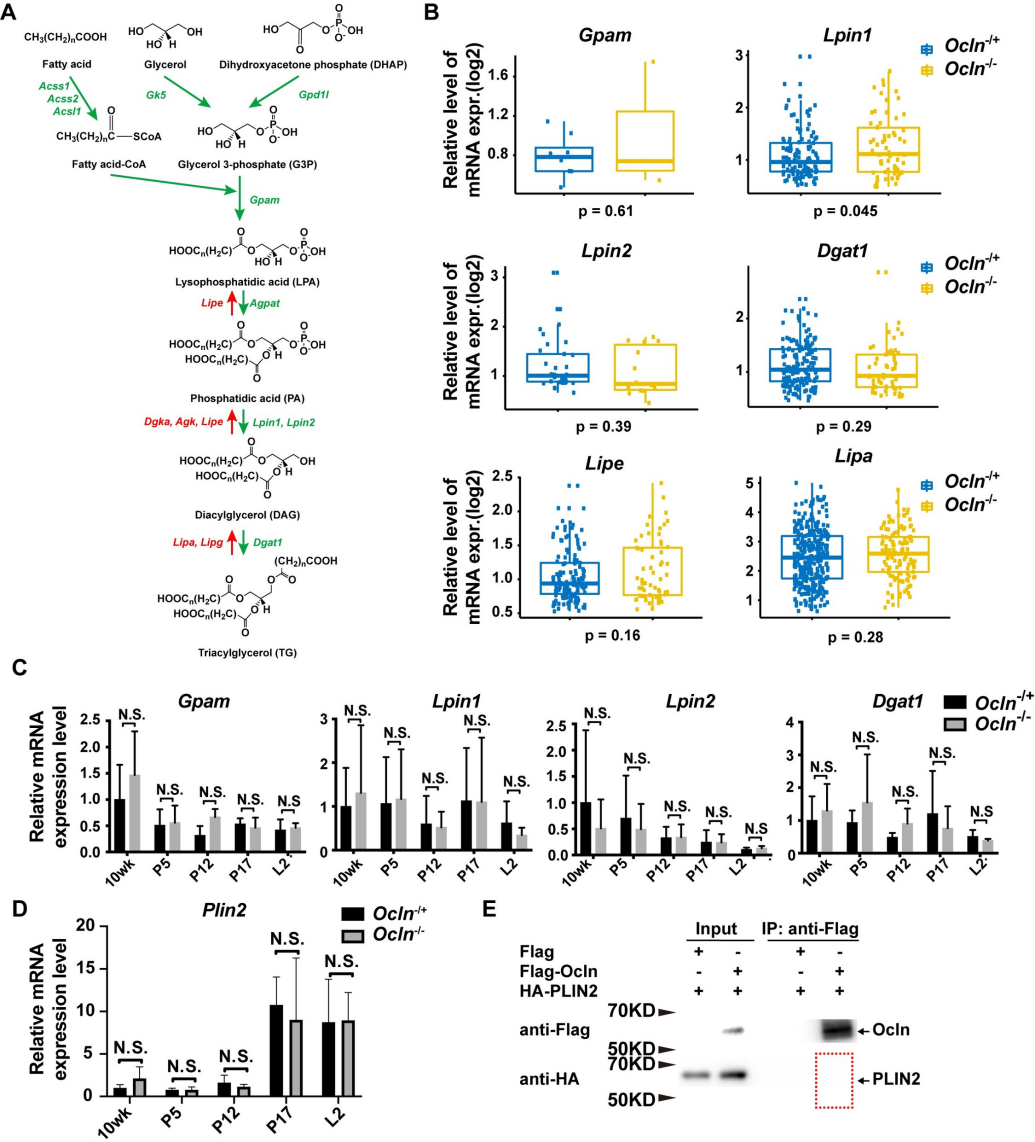
Triacylglycerol synthesis and degradation are relatively normal in *Ocln* null cells. (**A**) Overview diagram of de novo TG synthesis and the enzymes involved. (**B**) Relative gene expression levels of some of the genes in the TG synthesis pathway based on data from the scRNA-seq dataset (see Materials and methods). Each dot indicates the expression level based on log2 of the genes in a single cell. (**C**) Levels of mRNA expression as detected by qPCR of several key TG synthesis genes at the 10-wk, P5, P12, P17, and L2 stages. Values were normalized against *actin* expression, and gene expression at 10 wk of age was set as the base value against which other stages were compared. Graph shows mean ± SD. (**D**) Levels of *Plin2* mRNA expression as detected by qPCR at the 10-wk, P5, P12, P17, and L2 stages. The number of female mice at each stage used were: *Ocln*^−/+^ (*n =* 3) and *Ocln*^−/−^ (*n* = 3). (**E**) Immunoprecipitation assay to determine protein binding between OCLN and PLIN2. OCLN was tagged by Flag protein, whereas PLIN2 was tagged by HA. Antibody against Flag was used for immunoprecipitation, and antibody against HA was used for subsequent western blotting analysis. No binding of PLIN2 to OCLN was detected in this assay. Note that the individual numerical values that underlie the summary data here are listed in S1 Data file. HA, hemagglutinin; KD, kilodalton; L, lactation; *Ocln*, *Occludin*; P, pregnancy; PLIN2, Perilipin-2; qPCR, quantitative PCR; scRNA-seq, single-cell RNA sequencing; TG, triglyceride.

The increase in LD size in *Ocln* null alveoli at the L2 stage could result from an increase in TG synthesis or a decrease in its degradation in the mutant gland. We, therefore, examined the mRNA expression of the critical enzymes during TG synthesis and degradation (Fig 3A) [1]. In our previous study, we used single-cell RNA sequencing (scRNA-seq) technology and examined the transcriptomics of both control MECs and *Ocln* null MECs at the L2 stage [26]. Therefore, we mined the same scRNA-seq datasets and compared the mRNA expression of the critical lipid metabolic genes. We found that the mRNA expression of TG synthesis genes, including *Acsl1*, *Acss1*, *Acss2*, *Gk5*, etc., was similar in individual luminal cells of *Ocln* null and control mammary glands at the L2 stage (Figs 3B and S2A). Likewise, the mRNA expression of TG degradation genes, including *Lipe*, *Dgka*, *Agk*, *Lipa*, etc., was also similar between *Ocln* null and control mammary glands at this stage (Figs 3B and S2A).

Next, we sought to validate the results from the scRNA-seq data using quantitative PCR (qPCR). To identify a suitable housekeeping gene as an internal control for the qPCR analysis, we first examined *Actb* and *Gapdh* mRNA expression in our scRNA-seq dataset. We found that *Actb* and *Gapdh* mRNA expression was similar in both *Ocln* null and control mammary glands at the L2 stage (S3A Fig). Furthermore, when either *Actb* or *Gapdh* was used as an internal control for each other in qPCR assays, mRNA expression of the other gene was similar between *Ocln* null and control mammary glands. Likewise, *Cldn10*, whose expression was not affected by *Ocln* loss [26], was also found to have similar levels of mRNA expression irrespective of whether *Actb* or *Gapdh* was used as an internal control for qPCR (S3B Fig). These data suggest that both *Actb* and *Gapdh* were a good choice for internal controls for qPCR assays.

Therefore, we chose *Actb* as an internal control for our qPCR validation assays. We found that the mRNA expression of both TG synthesis and degradation genes were similar between *Ocln* null and control MECs at various developmental stages, including the 10-wk, P5, P12, P17, and L2 stages (Figs 3C and S2B).

A possibility exists where *Ocln* may affect the expression or function of *Plin2*, which coats the LDs and protects them from cytoplasmic lipases and thus lipolysis [1,9]. To address this possibility, we first analyzed mRNA expression of *Plin2* at several developmental stages, including the 10-wk, P5, P12, P17, and L2 stages. We found that *Plin2* mRNA expression was similar between *Ocln* null and control MECs at these stages (Fig 3D).

Next, we sought to determine whether OCLN could interfere with PLIN2 function by binding to it. We “tagged” both OCLN and PLIN2 at their N-termini using FLAG and hemagglutinin (HA) peptides, respectively. The fusion proteins were then subjected to co-immunoprecipitation (co-IP) assays to test their potential binding. We found that OCLN did not bind to PLIN2 in this assay (Figs 3E and S4A).

Finally, we determined whether OCLN and PLIN2 could colocalize in the living cells, which could also show their potential binding or interactions. Thus, we fused OCLN and PLIN2 proteins at their N-termini with a green fluorescent protein (GFP) and a mCherry protein, respectively, which were then introduced into primary MECs using lentiviral vectors. Using fluorescence confocal microscopy, we found OCLN did not colocalize with PLIN2 protein (S4B Fig; S1 Table, S1 Movie).

Taken together, the data suggest that the increase in LD size in *Ocln* null alveoli at the L2 stage unlikely results from an increase in TG synthesis or a decrease in TG degradation in the mutant gland.

### Lipid droplets grow normally but fail to be secreted by *Ocln* null cells

**Fig 4:**
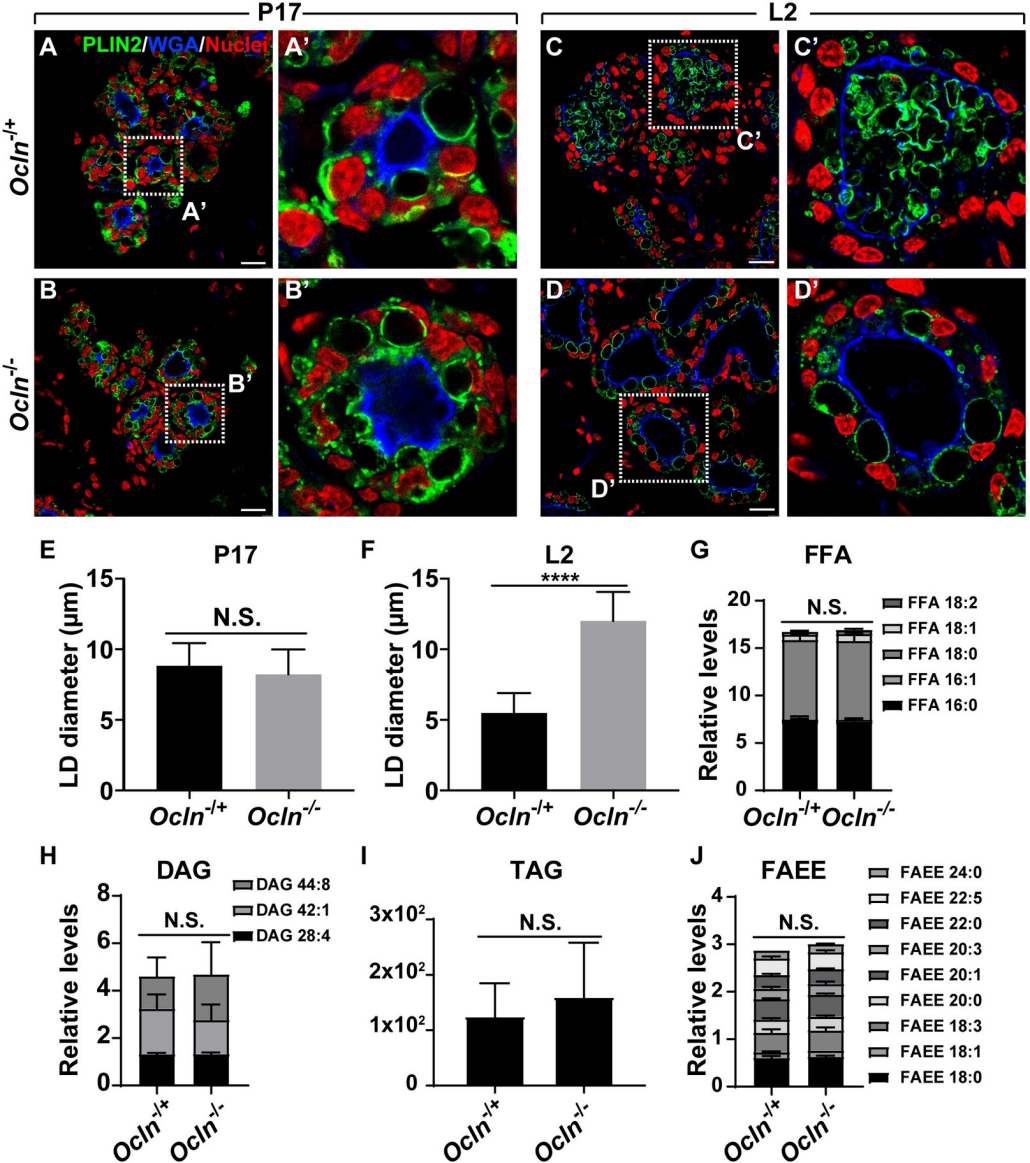
Lipid droplets grow normally but failed to be secreted by *Ocln* null cells. (**A-D’**) LDs as revealed by PLIN2 immunofluorescence (green) on mammary epithelia of *Ocln*^−/+^ and *Ocln*^−/−^ mice at the P17 (**A**, **B**, *Ocln*^−/+^, *n =* 3; *Ocln*^−/−^, *n* = 5) and the L2 stages (**C**, **D**, *Ocln*^−/+^, *n* = 4; *Ocln*^−/−^, *n* = 5). WGA staining (blue) marks the apical surface of the alveoli and is a demarcation of the lumen. Note that LDs are intracellular at the P17 stage. By the L2 stage (**C**-**D’**), they are secreted into the alveolar lumen in the form of MFGs in control glands by L2 (**C**, **C’**), whereas much less is observed in *Ocln* null glands (**D**, **D’**). Samples were counterstained with the nuclear dye DAPI (red). Insets show a close-up view of the alveolar epithelium. Scale bars: 20 μm. (**E, F**) Statistical analysis of LD sizes in *Ocln*^−/+^ and *Ocln*^−/−^ mammary gland alveoli at the P17 (**E**) and L2 (**F**) stages are shown. *t* test was used; ****P < 0.0001. (**G-J**) Relative levels of various lipid metabolic components, including FFAs (**G**), DAG (**H**), TAG (**I**), and FAEEs (**J**) from *Ocln* control and null mammary glands. The number of L2 female mice used were: *Ocln*^−/+^ (*n* = 3) and *Ocln*^−/−^ (*n* = 3). Note that the individual numerical values that underlie the summary data here are listed in S1 Data file. DAG, diacylglycerol; FAEE, fatty acid ethyl ester; FFA, free fatty acid; LD, lipid droplet; MFG, milk fat globule; *Ocln*, *Occludin*; PLIN2, Perilipin-2; TAG, triacylglycerol; WGA, wheat germ agglutinin.

Next, we used confocal microscopy to examine whether LDs in *Ocln* null MECs became larger than normal before or after the start of lactation. Using PLIN2 immunofluorescence, we found that many LDs, with an average diameter of approximately 9 μm, were present in the control alveoli at the P17 stage (Fig 4A, 4A’ and 4E). Interestingly, although milk proteins have been secreted into the lumen at the P17 stage [26], we did not observe any MFGs in the alveolar lumen by this stage (Fig 4A, 4A’ and 4E). By the L2 stage, MFGs were readily observed in the alveolar lumen, with a concurrent reduction of the number of intracellular LDs in the control epithelium (Fig 4C and 4C’). At this stage, the LDs, with an average diameter of approximately 5 μm, were smaller than those at the P17 stage (Fig 4C, 4C’ and 4F).

As in the control, LDs were also prominent in the *Ocln* null alveoli with a similar average diameter as that observed in the control alveoli (Fig 4B, 4B’ and 4E). However, by the L2 stage, LDs in the mutant alveoli were much larger than normal with an average diameter of around 13 μm. Unlike in the control alveoli where most lipids existed in the form of MFGs, in the *Ocln* null alveoli, they remained as intracellular LDs that were retained in the epithelium (Fig 4C–4D’).

Together, the results confirmed that there was an LD secretion defect in the *Ocln* null epithelium. The observation that LDs were larger than normal was most likely a secondary defect because of their inability to be efficiently secreted and, as a result, continued to grow inside luminal cells of the *Ocln* null epithelium. Consistent with this conclusion, we found that the levels of various lipid metabolic components, including free fatty acids (FFAs), diacylglycerol (DAG), triacylglycerol (TAG), and fatty acid ethyl esters (FAEEs) were indistinguishable between *Ocln* control and null mammary glands (Fig 4G, 4H, 4I and 4J), further confirming that TG biochemistry is relatively normal in the mutant glands.

### OCLN localizes to membranes of lipid droplets and milk fat globules

**Fig 5:**
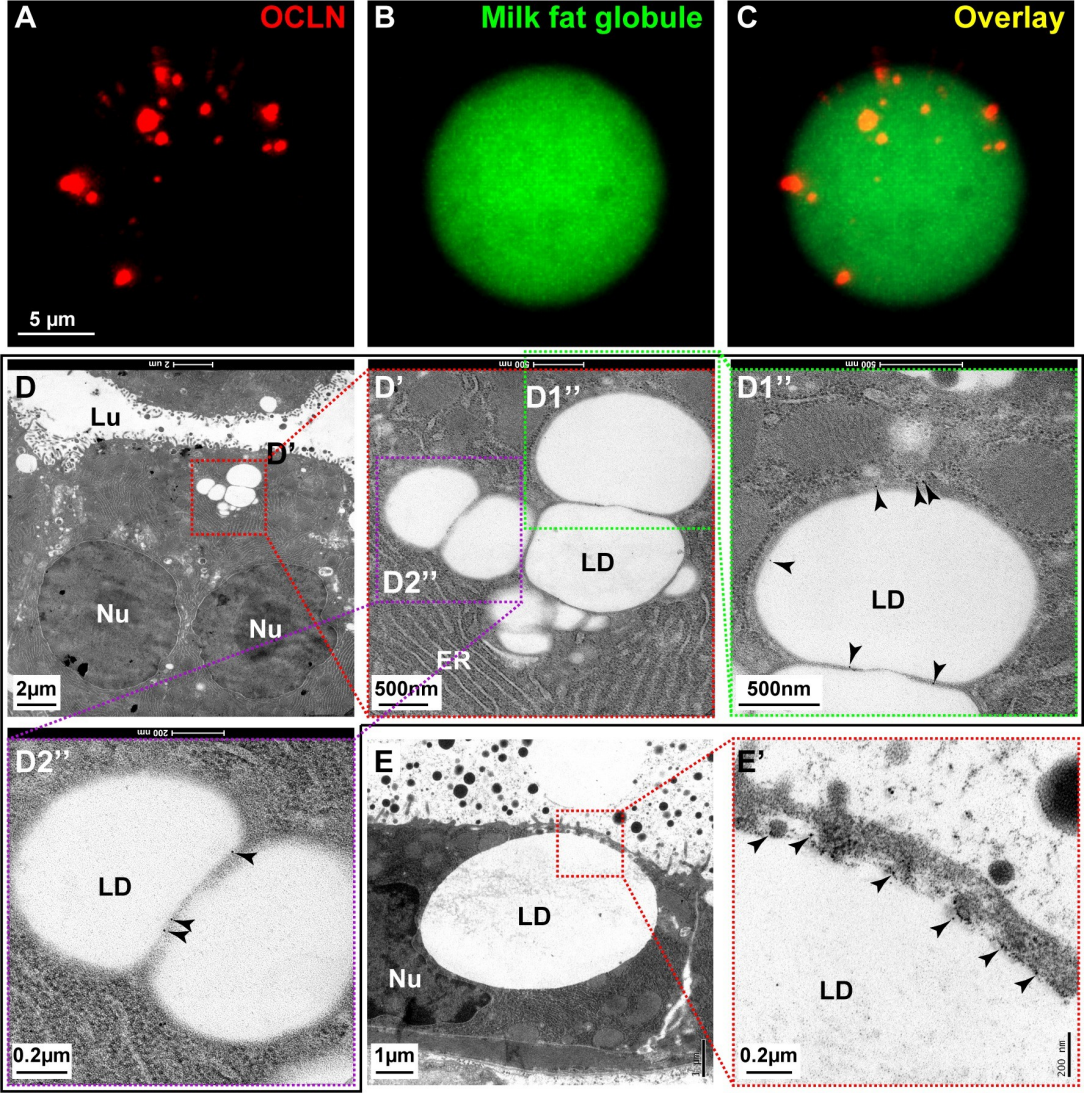
OCLN localizes to membranes of lipid droplets and milk fat globules. (**A-C**) Immunofluorescence of OCLN protein (red, **A**) on milk fat droplets, revealed by Bodipy (green, **B**) and overlay (**C**). Scale bar, 5 μm. Samples were taken from female mice at the L3 stage. A total of 659 LDs were examined. (**D**-**E’**) Localization of OCLN protein in luminal epithelial cells as detected by immunoelectron microscopy at the L2 stage. Primary antibodies against OCLN were visualized by a secondary antibody coupled to gold particles (black arrowheads). Areas in red or green-dotted boxes indicate close-up views. (**D’**, **D1”**-**D2”**) are progressive close-up views of LDs in (**D**) and (**D’**), respectively. (**E**, **E’**) indicate a close juxtaposition of an LD and apical plasma membrane. Scale bars are as indicated. ER, endoplasmic reticulum; LD, lipid droplet; Lu, lumen; Nu, nucleus.

OCLN may regulate LD secretion directly or indirectly. If OCLN is present at one or more places of the LD biosynthesis and secretion route, then the likelihood that OCLN plays a direct role in lipid secretion is greater than otherwise when it is absent. Using immunofluorescence, we found that OCLN was indeed present on MFGs in the milk (Fig 5A–5C). When ultrathin mammary gland tissue sections from the L2 stage were examined using immuno-EM, we found that OCLN, as shown by gold particles, was on the LD surfaces (Fig 5D–5D2”). Moreover, OCLN was also present at the contact sites between the mature LD, as judged by both its diameter and apical localization, and the plasma membrane (Fig 5E and 5E’).

Together, the data indicate that OCLN is present along the routes of LD development and secretion, suggesting that it plays a direct role in LD metabolism.

### OCLN binds to and colocalizes with lipid secretion regulators BTN1a1 and XOR

**Fig 6:**
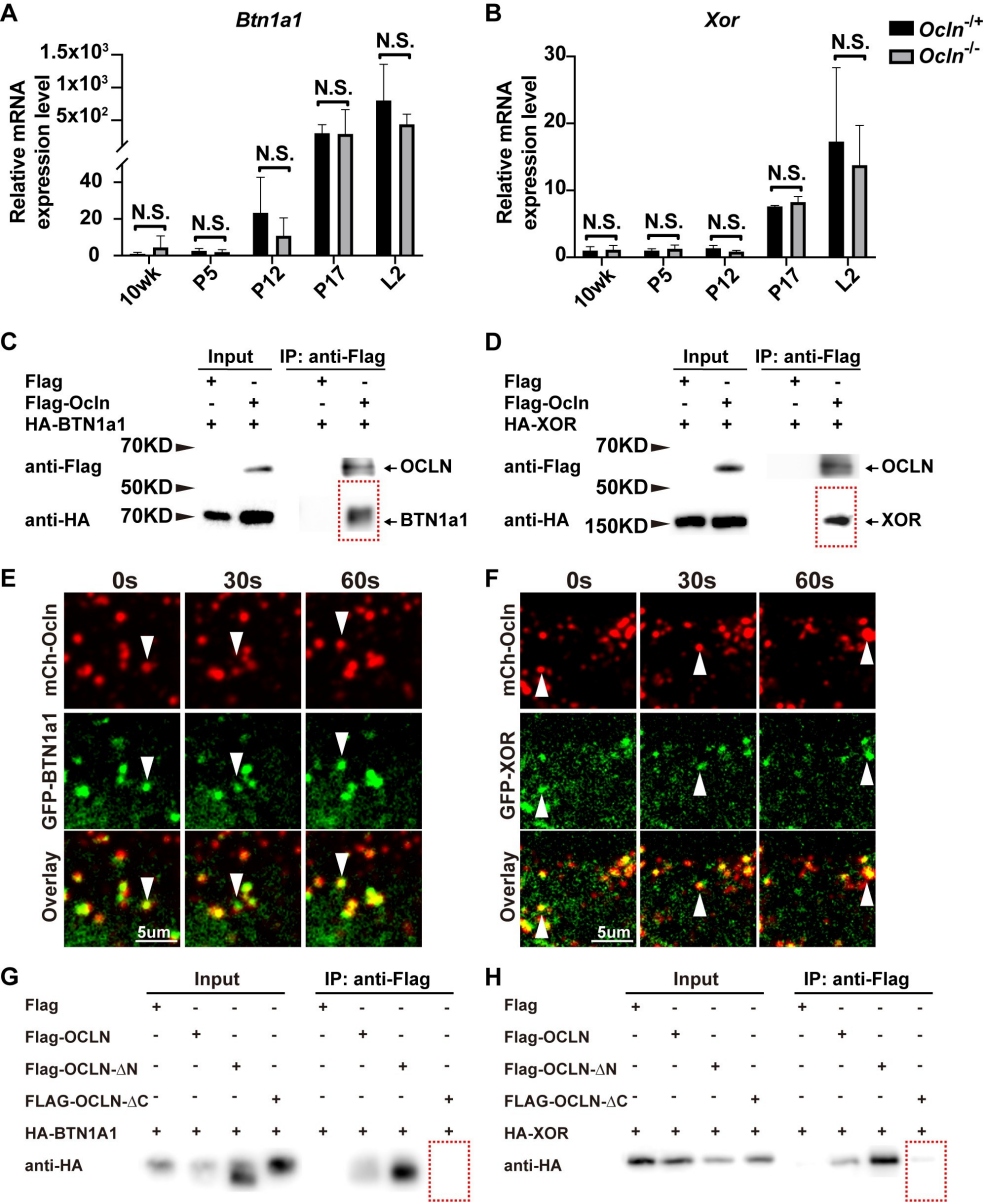
OCLN binds to and colocalizes with lipid secretion regulators BTN1a1 and XOR. (**A**, **B**) Levels of mRNA expression as detected by qPCR of LD secretion regulators *Btn1a1* (**A**) and *Xor* (**B**) in mammary gland epithelial cells at the 10-wk, P5, P12, P17, and L2 stages. Values were normalized against *actin* expression, and gene expression at 10 wk of age was set as the base value against which other stages were compared. Graph shows mean ± SD. The number of female mice at each stage used were: *Ocln*^−/+^ (*n =* 3) and *Ocln*^−/−^ (*n* = 3). (**C**, **D**) Protein binding between OCLN and BTN1a1 (**C**) and between OCLN and XOR (**D**) as detected by co-IP assays. OCLN was tagged by Flag protein, whereas BTN1a1 and XOR were tagged by HA. Antibody against Flag was used for immunoprecipitation, and antibody against HA was used for subsequent western blotting analysis. (**E**, **F**) Time course of localization of OCLN and BTN1a1 (**E**) or XOR (**F**) as detected by fluorescent microscopy. GFP was fused in-frame with OCLN at the N-terminus, whereas mCherry was fused in-frame with BTN1a1 (**E**) or XOR (**F**). White arrowheads mark OCLN and BTN1a1 (**E**) and XOR (**F**) particles over the time course of observation. Note that 54% and 44% of OCLN particles (**S1 Table**) colocalized with BTN1a1 (**E**) and XOR (**F**) particles, respectively. A total of 19 GFP-OCLN and mCherry-BTN1a1 double-positive cells and 28 GFP-OCLN and mCherry-XOR double-positive cells were examined in this experiment. (**G**, **H**) Protein binding between OCLN truncations and BTN1a1 (**G**) and between OCLN truncations and XOR (**H**) as detected by co-IP assays. OCLN truncations were tagged by Flag protein, whereas BTN1a1 and XOR were tagged by HA. Antibody against Flag was used for immunoprecipitation, and antibody against HA was used for subsequent western blotting analysis. Note that the individual numerical values that underlie the summary data here are listed in S1 Data file. co-IP, co-immunoprecipitation; GFP, green fluorescent protein; HA, hemagglutinin; KD, kilodalton; L, lactation; LD, lipid droplet; P, pregnancy; qPCR, quantitative PCR.

BTN1A1 and XOR are essential regulators of LD secretion that are thought to be directly involved in the juxtaposition of LD and the plasma membrane [5,6]. Therefore, we decided to examine the possibility that OCLN may participate in LD secretion by regulating Btn1a1 and Xor function. To this end, we first determined whether *Ocln* might regulate mRNA expression of *Btn1a1* and *Xor*. Using qPCR, we found that the mRNA expression of neither *Btn1a1* nor *Xor* was affected by *Ocln* loss at several stages of mammary gland development, including the 10-wk, P5, P12, P17, and L2 stages (Fig 6A and 6B). These data thus suggest that the cause of LD secretion failure was not due to abnormal expression of *Btn1a1* and *Xor*.

Alternatively, OCLN may bind to BTN1a1 and XOR to form a multiplayer complex, which is then required for LD secretion. To test whether OCLN is bound to BTN1a1 and XOR, we “tagged” OCLN and BTN1a1 or XOR proteins at their N termini with FLAG and HA peptides, respectively. Tagged proteins were then subjected to co-IP to test their potential binding. We found that OCLN bound to both BTN1a1 and XOR in this assay (Fig 6C and 6D). These in vitro data thus show that OCLN could bind to BTN1a1 and XOR.

Next, we sought to examine whether OCLN could bind to BTN1a1 and XOR proteins in vivo using the above colocalization assay. To this end, we fused OCLN and BTN1a1 or XOR proteins at their N-termini with mCherry and GFP, respectively. Lentiviral constructs expressing these fusion proteins were then used to transfect primary MECs. Transfected MECs were observed under fluorescence confocal microscopy. We found that OCLN frequently colocalized with BTN1a1 and XOR proteins, and they often migrated together inside the cells (Fig 6E and 6F, S1 Table, S2 and S3 Movies).

Finally, we wanted to examine whether OCLN binding to BTN1a1 and XOR depended on the N-terminus or C-terminus cytoplasmic domain of OCLN [22,27]. To this end, we tagged the N- or C-terminus truncation of OCLN with FLAG peptides. Tagged OCLN truncations were then introduced into 293 cells via transfection of transient expression constructs, followed by co-IP to test their potential interactions with BTN1a1 and XOR, which were tagged with the HA peptides. We found that the removal of the C-terminus of OCLN almost completely abrogated its ability to bind either BTN1a1 or XOR, while the removal of the N-terminus of OCLN did not have such an effect. These data suggest that the C-terminus is an essential functional component of OCLN during the regulation of lipid secretion via binding to BTN1a1 and XOR.

Taken together, the above data show that OCLN localizes to LDs and could directly participate in secretion by binding to BTN1a1 and XOR in MECs in a C-terminus–dependent manner.

### OCLN is a phosphorylation target of Src kinase during lipid secretion

Previous studies show that OCLN C-terminus contains multiple phosphorylation sites essential for its function [22,27]. We, therefore, suspected that OCLN might be a phosphorylation target of Src kinase, whose loss causes lactation failure and retainment of large LDs in MECs similar to OCLN [21,26]. First, however, we wanted to confirm that Src is indeed required for LD secretion. To this end, we treated female mice on the L1 stage for 24 h with either Src inhibitor 1 or the vehicle control and examined whether LDs could be secreted into the alveolar lumen. As expected, we found that MFGs were secreted into the lumen on L2 in the mammary glands of mice treated with the vehicle control (S5A Fig). By contrast, large intracellular LDs were present in the MECs, and MFGs were absent in the mammary glands of mice treated with Src inhibitor 1 (S5B and S5C Fig). The results thus confirmed that Src is required for LD secretion.

**Fig 7:**
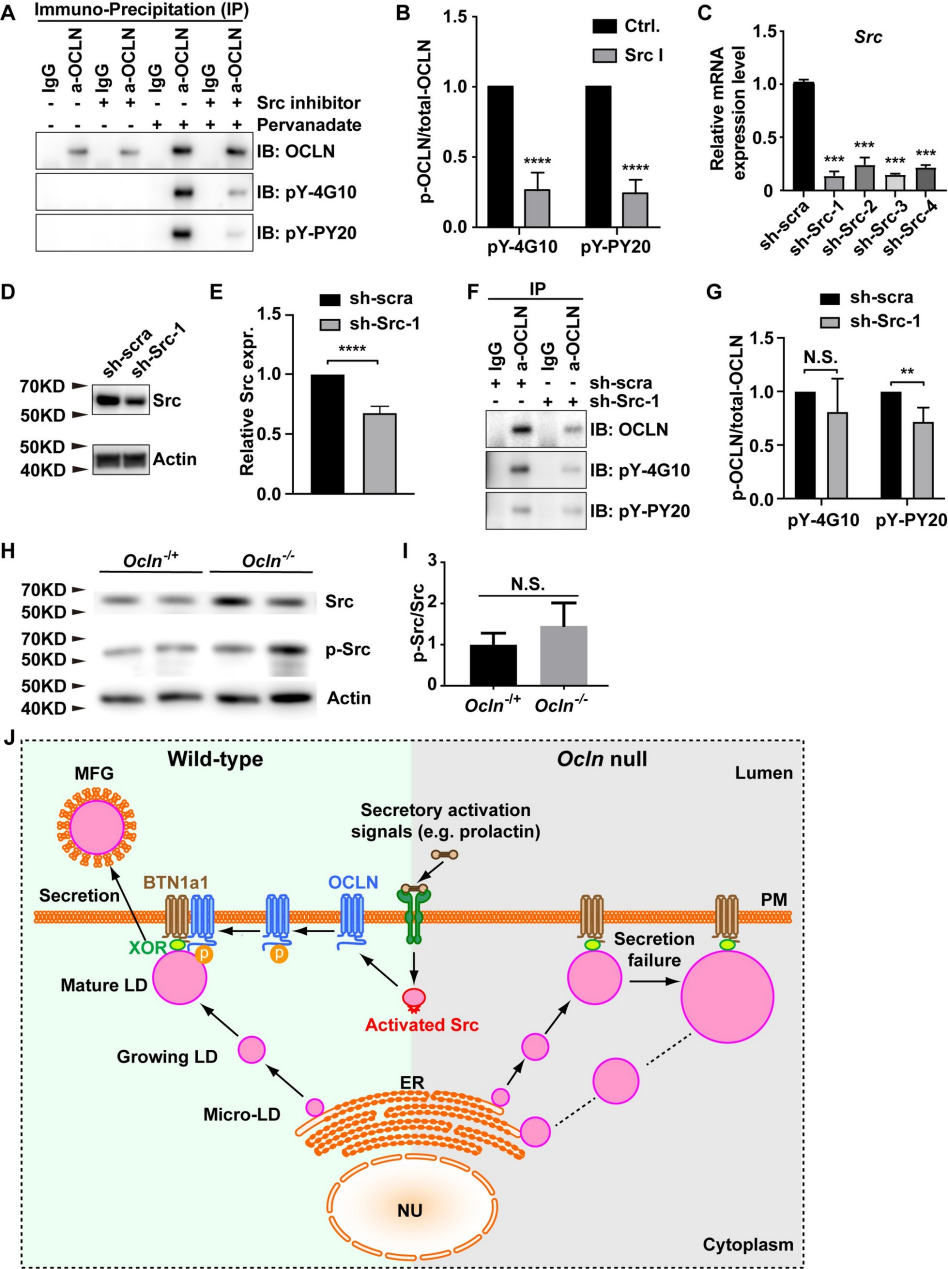
OCLN is a phosphorylation target of Src kinase during lipid secretion. (**A**, **B**) Effects of Src inhibitor 1 treatment on OCLN phosphorylation as detected by western blotting. (**A**) Samples were precipitated by antibodies indicated, followed by blotting using 2 antibodies against tyrosine phosphorylation sites. (**B**) Quantification shows Src inhibitor 1 caused approximately 75% reduction of OCLN phosphorylation using both pY-4G10 and pY-PY20 antibodies. Note that Src inhibitor 1 treatment specifically reduced phosphor-OCLN level but did not affect the total OCLN protein level. (**C-G**) Effects of blocking Src kinase activity by shRNA on OCLN phosphorylation as detected by western blotting. (**C**) Knockdown efficiency of 4 different shRNAs against Src as measured by mRNA expression levels. (**D**) Knockdown efficiency of sh-Src-1 as detected by western, which saw an approximately 30% reduction of Src protein expression level as measured by quantifying band density (**E**). (**F**, **G**) Levels of OCLN phosphorylation as a result of sh-Src-1 expression. Note that, unlike Src inhibitor 1, sh-Src-1 reduced total OCLN expression level (**F**). Thus, its effects on phosphor-OCLN were measured against the changes of total OCLN (**G**). After normalization, only the reduction detected by pY-PY20, but not pY-PY20, was considered to be statistically significant. (**H**, **I**) Protein expression of pSrc and total Src as detected by western blotting. Internal control was Actin (**H**). Relative levels of active Src as measured by the ratio of pSrc and total Src (**I**). **, *P* < 0.01; ***, *P* < 0.001; ****, *P* < 0.0001. (**J**) Schematic diagram of the lipid secretion process, which depends on Src kinase activation and OCLN function, in the mammary gland. In the *Ocln*^−/+^ control gland, signals of secretory activation, for example, prolactin upregulation, activate Src kinase. Src in turn phosphorylates and likely activates OCLN. This presumably leads to OCLN binding to BTN1a1 and XOR and promotes mature LDs to secrete to the outside of mammary cells. In mutant milk-producing cells, loss of *Ocln* function results in failed lipid secretion despite secretory activation. As a result, LDs build up along the biogenesis and secretion route, leading to enlarged LDs in *Ocln* mutant mammary glands. Note the individual numerical values that underlie the summary data here are listed in S1 Data file. ER, endoplasmic reticulum; LD, lipid droplet; MFG, milk fat globule; Nu, nucleus; PM, plasma membrane; pSrc, phosphorylated Src.

To determine whether OCLN is a phosphorylation target of Src kinase, we examined whether loss of Src function, using either Src inhibitor 1 or shRNA-mediated knockdown, affected OCLN phosphorylation. We found that Src inhibitor treatment did not affect OCLN protein expression levels but greatly reduced OCLN phosphorylation by over approximately 75% in HC11 cells (Fig 7A and 7B).

Next, we designed 4 shRNAs, including sh-Src-1, 2, 3, and 4 targeting different regions of the *Src* mRNA sequence. We found that they reduced *Src* mRNA expression levels by approximately 80% to 90% when compared to a control shRNA with a scrambled sequence (Fig 7C). We chose sh-Src-1, which among them caused the greatest Src mRNA reduction at approximately 90% (Fig 7C), to knock down *Src*. Interestingly, at the protein expression level, sh-Src-1 only reduced Src by approximately 30% in HC 11 cells (Fig 7D and 7E). However, HC11 cells expressing sh-Src-1 showed a significantly reduced level of OCLN phosphorylation at approximately 30% when pY-PY20 antibody was used (Fig 7F and 7G). While pY-4G10 also detected a decreased OCLN phosphorylation level in sh-Src-1 cells, the decrease was not significant (Fig 7G), presumably because that the sensitivity or specificity of these 2 phosphor-antibodies were different. Together, the data from both inhibitor and shRNA experiments demonstrate that OCLN is a phosphorylation target of Src kinase during lipid secretion.

Although the above data suggest that OCLN functions downstream of Src kinase, it is possible that there exists a feedback loop between them and, as such, OCLN may in turn regulate Src function as well. To test this possibility, we examined whether *Ocln* loss affected the levels of Src protein expression and its phosphorylation. We found that both Src protein and its phosphorylation levels were similar between *Ocln* null and control MECs (Fig 7H and 7I). These data suggest that Src kinase was not downstream of OCLN function and that the failure of LD secretion phenotype in *Ocln* null alveoli was not due to compromised Src function.

Finally, we determined whether *Ocln* regulated several other key components of LD secretion, including transcription factors *Th-POK* and *Cidea*, and an RNA-binding protein *TDP-43*. To this end, we used qPCR and examined mRNA expression of these genes at several stages of mammary gland development, including at the 10-wk, P5, P12, P17, and L2 stages. We found that none of these genes showed abnormal mRNA expression in *Ocln* mutant MECs than in control MECs (S5D–S5F Fig). These data thus suggest that the cause of LD secretion failure in *Ocln* null mammary glands was not due to the abnormal expression of the above key LD secretion regulators.

Taken together, the above data show that loss of *Ocln* function does not affect the expression of known LD secretion regulators. However, OCLN is a phosphorylation target of the lipid secretion regulator Src kinase, suggesting that the failure of LD secretion in *Src* null mammary glands resulted from the loss of OCLN phosphorylation and activation.

## Discussion

LDs are essential organelles in eukaryotes. However, regulation of LD dynamics, especially its secretion, has remained largely unknown. In the current study, we report that mice lacking *Ocln* show defective lipid metabolism. We found that in mammary glands lacking *Ocln*, LDs were larger than normal at multiple sites along its biogenesis and secretion pathway. The defects were not a result of abnormal lipid synthesis or degradation; rather, they resulted from a failure of LD secretion during lactation. Consistent with an essential role in lipid secretion, OCLN is located on LD membranes and bound to essential regulators including BTN1a1 and XOR. We show that OCLN is a phosphorylation target of Src kinase, whose loss causes a failure of lactation and lipid secretion.

Together, our results support a model in which *Ocln* promotes LD secretion by binding to BTN1a1 and XOR downstream of Src kinase signaling. Specifically, we show that the lipid secretion process depends on Src kinase activation and OCLN function in the mammary gland. In the *Ocln*^−/+^ control gland, signals of secretory activation, for example, prolactin upregulation, activate Src kinase (Fig 7J). Src in turn phosphorylates and likely activates OCLN, which presumably leads to binding to BTN1a1 and XOR and promotes mature LDs to secrete to the outside of mammary cells. In mutant milk-producing cells, loss of *Ocln* function results in lipid secretion failure despite secretory activation. As a result, LDs build up along the biogenesis and secretion route, leading to enlarged LDs in *Ocln* mutant mammary glands (Fig 7J).

### OCLN promotes lipid secretion by binding to BTN1a1 and XOR

Our data show that mammary glands lacking *Ocln* were defective in lipid secretion, suggesting that it is required for this important process. The observation that OCLN was located on the membranes of both the MFGs and, more importantly, the LDs further supports that *Ocln* plays a direct role in regulating LD secretion. Indeed, results from both the co-IP and immunofluorescent colocalization assays show that OCLN was bound to the LD secretion regulators BTN1a1 and XOR. Like OCLN, both BTN1a1 and XOR are required for LD secretion [19,20]. Together with the observation that BTN1a1 and XOR are located on the plasma membrane and/or the LD surfaces, it has been proposed that a complex containing both BTN1a1 and XOR is required at the plasma membrane and LD interface is essential for facilitating LD secretion [5,6]. Our results are consistent with this model and further suggest that the complex also contains OCLN as an additional player essential for this biological process.

It is well characterized that PLIN2, as a resident coat protein on the LD surface, promotes LD growth by protecting it from cytoplasmic lipases [1,9]. However, a model has been proposed that PLIN2 forms a complex with BTN1a1 and XOR and is essential for LD secretion [28,29]. Interestingly, loss-of-function studies show that PLIN2 is not required for LD secretion [30,31]. This is consistent with our data, based on both co-IP and colocalization studies, showing that OCLN does not bind to PLIN2. Together, our data do not support the proposed role of PLIN2 in LD secretion but agree with its conventional function in being a shield protein that protects LD growths.

At present, it remains unclear the detailed mechanism by which OCLN promotes LD secretion. As we recently showed, OCLN promotes milk protein secretion, which involves a conventional SNARE-dependent membrane fusion event between secretory vesicles and the plasma membrane [26]. Considering that OCLN functions in these 2 distinct secretory processes, one likely possibility is that OCLN regulates a common step shared by both LD and milk protein secretion, rather than a step unique only to one of these 2 processes. In this light, it is tempting to speculate that OCLN may be essential for bringing close, or “docking,” of the “cargos,” whether secretory vesicles or LDs and the plasma membrane. Future studies, especially if LD secretion could be recapitulated in vitro, should hopefully be able to test this possibility and shed important new light on OCLN function in LD secretion.

Finally, based on its amino acid sequence, OCLN has previously been predicted as a conventional four-pass transmembrane protein [22,27]. While this prediction is consistent with OCLN localization on the plasma membrane and secretory vesicles [26], it cannot explain how OCLN could locate on the single-layered phospholipid LD membrane as our immuno-EM results showed (Fig 5), or in the cytoplasm and centrosomes as reported by others, which all have important functional consequences [24,25]. Interestingly, a similar precedent has already been reported. Specifically, BTN1a1, a single-pass transmembrane protein based on its amino acid sequence [32], has also been convincingly shown to locate on the LD monolayer by FRIL electron microscopy [5,33]. In both of these 2 cases with OCLN and BTN1a1, a final resolution of this apparent paradox will likely have to wait until their structures under physiological conditions become clear to us.

### Secretion of LDs and milk proteins are independent processes

An in vitro system in which LDs could be reliably secreted would allow us to directly interrogate the mechanism by which OCLN regulates LD secretion and thus shed important new light on this poorly understood process. It was disappointing that our results show that, although LD formation and growth could be recapitulated using the 3D in vitro assays, lipid secretion was not achievable because LDs were never observed in the lumen regardless of whether pharmacological means to raise Ca^2+^ concentration were used under the published conditions. The most likely explanation for the apparent contradiction between our results and previous studies [12,34] is that LD presence in the lumen had never been validated as carefully as we did here, where both appropriate genetic tools, including the *R26R*^mTmG^ mice, and better imaging techniques, including confocal microscopy and 3D reconstructions, were used.

It has previously been observed that mature LDs are often in close contact with and, in many cases, surrounded by secretory vesicles at the apical membrane of luminal cells, prompting a theory that suggests that lipid secretion in the mammary gland may depend on milk protein secretion [7]. Our data do not support this theory. Specifically, we found that LDs grew during pregnancy and were not secreted until lactation (Fig 4). By contrast, our previous results show that milk proteins are secreted, as evident from their presence in the alveolar lumen, during pregnancy [26]. Thus, the data show the kinetics of LD secretion and protein secretion are different. Consistent with these results, LD size was normal in *Ocln* null during pregnancy and did not become obvious until after lactation; by contrast, defective milk protein section was already evident during late pregnancy in the mutant mice.

Moreover, it is well known that milk proteins are secreted using both a constitutive and, especially, a regulated secretion pathway [35]. Regulated secretion is triggered by Ca^2+^ as a second messenger [36]. In the presence of increased Ca^2+^ ions, for example, when stimulated by ionomycin, which is thought to increase Ca^2+^ concentration, protein secretion is also increased. Interestingly, although protein secretion is effective in in vitro cultures and can be stimulated by ionomycin [35], LD secretion was not triggered. Together, these results show that protein and LD secretion are 2 independent processes that are differentially regulated. However, this does not exclude the possibility that protein secretion and LD secretion share some common players. Indeed, our studies show that OCLN is required for both processes, as is Src kinase. As we understand more about both of these 2 processes, we should have a better understanding of what common and different regulators they use.

Finally, it is intriguing that, although LDs are larger than normal in *Ocln* null cells at the L2 stage, lipid levels in the mutant glands are similar to that in the control glands at this stage. It is possible that there exists a hitherto unknown mechanism by which the mammary gland senses the amount of lipids secreted into the lumen and “adjusts” it to its “normal” physiological levels. Alternatively, and not mutually exclusively, the relatively normal lipid levels in the mutant gland may be a secondary consequence of a concomitant reduction of protein secretion by the *Ocln* null epithelial cells [26]. We await future studies to yield new insights into these intriguing possibilities.

## Materials and methods

### Mouse strain

Mice carrying the *Ocln* allele were genotyped as previously described [37]. Mice were housed and maintained according to regulations from ShanghaiTech University’s Institutional Animal Care and Use Committee (IACUC# 20200713003).

### Mammary gland preparations, sectioning, staining, and imaging

Mammary glands were harvested and mounted on glass slides. They were stained with Carmine Red, cleared in Histoclear [38], and photographed using a Zeiss Lumar dissection scope, as described previously [39]. Where necessary, mammary glands were fixed and embedded in paraffin. They were then cut at 5 μm per section and stained in H&E staining solution. For frozen sections, mammary glands were harvested and fixed in 4% paraformaldehyde overnight at 4°C. Using a Leica cryostat, 10-μm frozen sections were cut. DIC microscopy on a converted Zeiss Z1 was used to visualize LDs.

### Immunofluorescence and confocal microscopy

Frozen sections of mammary glands were for immunofluorescence analysis. They were first blocked for 1 h at room temperature (RT) in PBS containing 10% goat serum and 0.2% Tween20, followed by incubation in primary antibodies for 1 h at RT or overnight at 4°C. The primary antibodies used in this study was the PLIN2 antibody (Novus, #NB110-40877, 1:100 dilution); the OCLN antibody (ThermoFisher, #40–6100, RRID AB_2533473). WGA (Alexa Fluor 488) was from eBioscience (#W11261, 1:1,000 dilution). Confocal microscopy was performed on a Leica SP8 STED confocal.

### Ultrathin section and immuno-electron microscopy

Very small pieces (approximately 1 mm^3^) of #4 mammary glands were fixed in 2% glutaraldehyde and 1% paraformaldehyde overnight. They were then fixed again in OsO4 for 2 h before embedding using the Embed 812 Kit (Electron Microscopy Sciences, Cat# 14120). Ultrathin sections were cut using a diamond knife and double-stained with uranyl acetate and lead citrate before the examination.

For immuno-electron microscopy, tissues were fixed in 0.2% glutaraldehyde and 4% paraformaldehyde before they were embedded in Epon 812. Ultrathin sections were then cut and sequentially treated with sodium periodate, 0.1% glycine, and 1% BSA before they were incubated with OCLN antibody for 48 h at 4°C. After washing, samples were incubated with goat anti-rabbit IgG secondary antibody, which was conjugated with 10 nm gold particles (Sigma, #G7402) at a 1:40 dilution for 2 h at RT. Samples were refixed with 1% glutaraldehyde, double-stained with uranyl acetate and lead citrate, then examined using a Joel JEM-1230 (Institute of Neurosciences, Academia Sinica) or a Talos L120C (ShanghaiTech University) transmission electron microscope at an accelerating voltage of 80 kV.

### Milk harvest and LC–MS analysis

Pups were removed from dams for 2 h to allow milk accumulation in the mammary glands. Dams were sedated using xylazine, IP delivered at a dose of 8 mg/kg. A single dose of oxytocin (0.5 IU) was given to the dam to stimulate milk release. Milk was collected using a capillary tube. A volume of 50 μl milk was used for LC–MS analysis, which was performed by Dr. Piliang Hao and Ms. Zhaomei Shi from the Bio-Mass Spectrometry Core Facility (BMSCF) in the School of Life Science and Technology at ShanghaiTech University. Briefly, lipids were extracted by adding 375 μl methanol to the milk sample and vortexed. A volume of 1,250 μl methyl-tert-butyl ether (MTBE) was added to the mixture, which was incubated for 1 h at RT in a shaker. Phase separation was induced by adding 313 μl MS-grade water, followed by centrifugation at 1,000 × *g* for 10 min. The organic (upper) phased was collected and dried in a vacuum centrifuge. Samples were then used for MS analysis.

### Bioinformatics analysis

The datasets were previously described and deposited at the National Center for Biotechnology Information Sequence Read Archive (SRA) (www.ncbi.nlm.nih.gov/sra/; accession nos. SRR10481973 and SRR10481974) [26]. Briefly, the Cell Ranger R kit was used for quality control and data filtering. Only cells with mRNA expression of at least 2,000 genes were used for analysis. The ggpubr R kit was used for the quantitative comparison of gene expression between Ocln null and control mammary cells. *t* test was used for statistical analysis.

### Quantitative real-time PCR

Total RNA was prepared from the whole mammary gland using Trizol reagents (Vazyme, R401-01). Equal amounts of RNA templates were reverse transcribed into cDNAs using HiScript II Q Select RT SuperMix for qPCR (Vazyme Biotech, R233). Then, cDNAs were used in qPCR reactions using ChamQ Universal SYBR qPCR Master Mix (Vazyme Biotech, Q711) with the BIO-RAD CFX Connect Real-Time Systems according to the manufacturer’s protocol. Relative expression levels were calculated using the comparative CT method. Data were normalized to the expression of 2 reference genes, including *Actb*, *Gapdh*. The primers used are listed in S2 Table.

### mRNA expression reduction using shRNA knockdown

The design of shRNAs was based on an online tool at ThermoFisher (https://rnaidesigner.thermofisher.com/rnaiexpress/sort.do). shRNA fragments were then cloned into a modified version of the pLKO.1-GFP vector (Addgene # 8453). The 293 cell line was used for construct transfection and lentiviral production using a standard protocol. Lentivirus was concentrated, titer determined, and was used to infect HC11 cells. Infected cells were selected using FACS and positive cells were then cultured for subsequent qPCR and western blotting assays. The shRNA sequences were as follows. scramble shRNA (sense, 5′-ACCTAAGGTTAAGTCGCCCTCG-3′; antisense, 5′-CGAGGGCGACTTAACCTTAGGT-3′); sh-Src-1 (sense, GCGGCTGCAGATTGTCAATAA; antisense, TTATTGACAATCTGCAGCCGC); sh-Src-2 (sense, GGCTGCAGATTGTCAATAACA; antisense, TGTTATTGACAATCTGCAGCC); sh-Src-3 (sense, GCTCATAGAAGACAACGAATA; antisense, TATTCGTTGTCTTCTATGAGC); sh-Src-4 (sense, GCCTAAATGTGAAACACTACA; antisense, TGTAGTGTTTCACATTTAGGC).

### Western blotting and co-immunoprecipitation (Co-IP) assays

Organoid or HC11 cell preparations were lysed in buffer containing 20 mM Tris–HCl (pH 7.4), 150 mM NaCl, 1 mM EDTA, 1 mM EGTA, 5 mM NaF, 1 mM orthovanadate, 10% (vol/vol) glycerol, 1% Triton X-100, 0.5% Nonidet P-40, 1 mM phenylmethylsulfonyl fluoride, 2 μg/mL leupeptin, and 10 μg/ml aprotinin. Samples were cleared with centrifugation at 13,500 × *g* at 4°C for 10 min. Protein concentrations were quantified with a Pierce BCA protein assay kit (Thermo Fisher Scientific), and 5 to 10 μg was resolved by SDS/PAGE under reducing conditions in regular Tris-glycine buffer on a gradient gel Supersep Ace 5% to 20% (Wak9). Proteins were electrically transferred in a wet tank to a PVDF membrane. After blocking with 5% BSA (sigma#WXBC3116V), target proteins were visualized using mouse anti-OCLN, rabbit anti-Src (Santa Cruz Biotech, #sc-271542), and rabbit anti-p-Src antibodies (Thermo Fisher, #71–6900). For quantification of protein expression, band density was measured using an Amersham Imager 680.

For Co-IP assays, the 293T line of cells expressing FLAG-OCLN/OCLN-ΔN/OCLN-ΔC and HA-PLIN2/BTN1a1/XOR were harvested, washed twice with PBS, then lysed in buffer containing 20 mM HEPES, 150 mM NaCl, and 1% NP40. Samples were coincubated with FLAG-tagged magnetic beads (Bimake, #B26101) O/N at 4°C. Beads were then washed 4 times with washing buffer before coincubated with 40 μl 2 X sample loading buffer to harvest FLAG-tagged proteins before they were subjected to western blotting analysis. The antibodies used were anti-FLAG (GNI, #GNI4110-FG-S) and anti-HA (Cell Signaling Technology, #2999). Band density was measured using an Amersham Imager 680.

For detection of Occludin tyrosine phosphorylation levels, HC11 cells were cultured in basic RPMI 1640 medium (Invitrogen, #C11875500CP), supplemented with 10% FBS, penicillin (5 U/mL) and streptomycin (5 μg/mL), insulin (5 μg/mL), and epidermal growth factor (10 ng/mL). Once cells became confluent, they were treated with or without 10 μM Src inhibitor 1 (Selleck, Cas#179248-59-0) for 48 h. The cells were treated with 1 mM pervanadate at 37°C for 10 min before harvest. Total proteins from the cells were extracted with NP-40 lysis buffer [50 mM Tris (pH 7.5), 150 mM NaCl, 1% NP-40, containing phosphatase inhibitor cocktail (Bimake, #B15002), protease inhibitor cocktail (Bimake, #B14001), and 0.2 mM activated Na3VO4]. The extract was centrifuged to remove undissolved particles, and the supernatant was used for immunoprecipitation. About 2 μg normal rabbit IgG (Cell Signaling, # 2729S) or rabbit anti-Occludin antibody (Invitrogen, #406100) were coupled on protein A/G plus-agarose (SantaCruz, #sc-2003) at 4°C for 12 h. Supernatants (1 mg of protein) were incubated with the coupled antibody for 16 h at 4°C. Immune complexes were then immunoblotted for anti-phosphotyrosine antibody (Merk, #05–321; SantaCruz, #sc-508) or anti-Occludin antibody (Novus #ab216327).

### Effects of Src inhibition on lipid secretion

Src inhibitor 1 (Selleck, Cas#179248-59-0) was dissolved using an in vivo formulation (5% DMSO, 40% PEG300, 5% Tween-80, 50% H2O) at 0.45 mg/mL and was administered by intraperitoneal injection. The dose was formulated so as to inject 0.05 mL of vehicle/10*g* of body weight. Experimental mice received a dose of 2.25 mg/kg body weight of Src inhibitor 1. The treatment started at 9 AM on L1, and the mammary glands were harvested 24 h later at 9 AM on L2. Mammary glands were then processed for cryo-sectioning and immunofluorescent staining.

### Colocalization assay

For the colocalization assay, HC11 cells overexpressing both mCherry-OCLN and EGFP-PLIN2/BTN1a1/XOR were cultured in the differentiation medium (DMEM/F12 containing 50 U/ml P/S, 5 μg/ml insulin, 50 μg/ml gentamycin, 1 μg/ml hydrocortisone, and 3 μg/ml prolactin) for 4 days and were then visualized on a Zeiss LSM980 confocal. ImageJ was used for analysis and statistical analysis of colocalization coefficients were calculated using the JACoP plugin.

## Supporting information

S1 Table Colocalization coefficients of OCLN and PLIN2, BTN1a1, and XOR. (DOCX)

S2 Table Primers used in qPCR. (DOCX)

S1 Fig Lipid droplets are larger than normal in Ocln mutant mammary glands at multiple sites along its biogenesis and secretion route. (A) Statistical analysis of LD sizes in Ocln^−/+^ and Ocln^−/−^ mammary gland alveoli at the L2 stage is shown. *t* test was used; *P < 0.05, **P < 0.001, ***P < 0.001. LD, lipid droplet; Ocln, Occludin.
(TIF)

S2 Fig Triacylglycerol synthesis and degradation are relatively normal in *Ocln* null cells. (A) Relative gene expression levels of some of the genes in the TG synthesis pathway are based on data from the scRNA-seq dataset. Each dot indicates the expression level based on log2 of the genes in a single cell. (B) Levels of mRNA expression as detected by qPCR of several key TAG synthesis genes at the 10-wk, P5, P12, P17, and L2 stages. Values were normalized against *actin* expression, and gene expression at 10 wk of age was set as the base value against which other stages were compared. Graph shows mean ± SD. The number of female mice at each stage used were: *Ocln*^−/+^ (*n =* 3) and *Ocln*^−/−^ (*n* = 3). L, lactation; *Ocln*, *Occludin*; P, pregnancy; qPCR, quantitative PCR; scRNA-seq, single-cell RNA sequencing; TAG, triacylglycerol; TG, triglyceride.
(TIF)

S3 Fig mRNA expression of both *Actb* and *Gapdh* was similar between control and null glands. (A) Relative gene expression level of *Actb* and *Gapdh* based on data from the scRNA-seq dataset. Each dot indicates the expression level based on log2 of the genes in a single cell. (B) Levels of mRNA expression as detected by qPCR at the L2 stage. Note that expression of *Actb*, *Gapdh*, and *Cldn10* was comparable between control and *Ocln* null glands, irrespective of whether *Actb* or *Gapdh* was used as an internal control. Graph shows mean ± SD. The number of female mice at the L2 stage used were: *Ocln*^−/+^ (*n =* 3) and *Ocln*^−/−^ (*n* = 3). L, lactation; P, pregnancy; qPCR, quantitative PCR; scRNA-seq, single-cell RNA sequencing.
(TIF)

S4 Fig OCLN does not bind to or colocalize with PLIN2. (A) Immunoprecipitation assay to determine protein binding between OCLN and PLIN2. OCLN was tagged by Flag protein, whereas PLIN2 or BTN1a1 was tagged by HA. Antibody against Flag was used for immunoprecipitation, and antibody against HA was used for subsequent western blotting analysis. No binding of PLIN2 to OCLN was detected in this assay (lane 7 marked with an asterisk). The association of BTN1a1 with OCLN was detected in the immunoprecipitation assay (lane 7 marked with an asterisk) and served as a positive control. (B) Time course of localization of OCLN and PLIN2 as detected by fluorescent microscopy. mCherry was fused in-frame with OCLN at the N-terminus, whereas GFP was fused in-frame with PLIN2. Statistical analysis shows that 2% of OCLN particles colocalized with PLIN2 (S1 Table). Scale bars: 2 μm. GFP, green fluorescent protein; HA, hemagglutinin; PLIN2, Perilipin-2.
(TIF)

S5 Fig OCLN is a phosphorylation target of Src kinase during lipid secretion. (A-C) Src inhibition impairs LD secretion. LDs as revealed by PLIN2 immunofluorescence (green) on mammary epithelia of control (A) and Src inhibitor-treated (B) mice at the L2 stage. WGA staining (blue) marks the apical surface of the alveoli and is a demarcation of the lumen. Note that LDs have been secreted into the alveolar lumen in the form of MFGs in the control glands. By contrast, while few MFGs are present in the lumen of Src inhibitor-treated glands, a buildup of large LDs are inside epithelial cells of these glands. Samples were counterstained with the nuclear dye DAPI (red). Scale bars: 10 μm. (C) Statistical analysis of LD sizes in control and Src inhibitor-treated mammary gland alveoli at the L2 stage are shown. Note that, while the percentages of small- and medium-sized LDs were indistinguishable between the control and experimental groups, the percentage of large LDs (>4 μm) was significantly more in the Src inhibitor group than in the control group. *t* test was used. (D-F) Levels of mRNA expression as detected by qPCR of LD secretion regulators, including *Th-POK* (D), *Cidea* (E), and *TDP-43* (F) in mammary gland epithelial cells at the 10-wk, P5, P12, P17, and L2 stages. Values were normalized against *actin* expression, and gene expression at 10 wk of age was set as the base value against which other stages were compared. Graph shows mean ± SD. The number of female mice at each stage used were: *Ocln*^−/+^ (*n* = 3) and *Ocln*^−/−^ (*n* = 3). L, lactation; LD, lipid droplet; MFG, milk fat globule; P, pregnancy; PLIN2, Perilipin-2; qPCR, quantitative PCR; WGA, wheat germ agglutinin.
(TIF)

S1 Movie Live imaging of mammary epithelial cells co-transfected with mCherry-OCLN and GFP-PLIN2. (MP4)

S2 Movie Live imaging of mammary epithelial cells co-transfected with mCherry-OCLN and BTN1a1. (MP4)

S3 Movie Live imaging of mammary epithelial cells co-transfected with mCherry-OCLN and XOR. (MP4)

S1 Data Individual numerical values that underlie the summary data displayed in Fig 1K, 1L; Fig 3B–3D; Fig 4E–4J; Fig 6A, 6B; Fig 7B, 7C, 7E, 7G and 7I; S1 Fig; S2A and S2B Fig; S3A and S3B Fig and S5C–S5F Fig. (XLSX)

S1 Raw image Original gel images of all Western Blotting results. (PDF)

## Abbreviations

co-IP: co-immunoprecipitation
DAG: diacylglycerol
DIC: differential interference contrast
ER: endoplasmic reticulum
FAEE: fatty acid ethyl ester
FFA: free fatty acid
GFP: green fluorescent protein
HA: hemagglutinin
H&E: hematoxylin and eosin
LD: lipid droplet
MEC: mammary epithelial cell
MFG: milk fat globule
*Ocln*: *Occludin*
PLIN2: Perilipin-2
qPCR: quantitative PCR
RT: room temperature
scRNA-seq: single-cell RNA sequencing
TAG: triacylglycerol
TEM: transmission electronic microscopy
TG: triglyceride
WGA: wheat germ agglutinin
