# Supplementary material for: Occludin is a target of Src kinase and promotes lipid secretion by binding to BTN1a1 and XOR
Source: PLoS Biol. 2022 Jan 18;20(1):e3001518. doi: 10.1371/journal.pbio.3001518 (PMC8797263; doi:10.1371/journal.pbio.3001518)
Supplement: S1 Table — (DOCX) [file pbio.3001518.s001.docx]

S1 Table. Colocalization coefficients of OCLN and PLIN2, BTN1a1, and XOR.

|  | Pearson's Coefficient | Overlap Coefficient | Manders' Coefficients | |
| --- | --- | --- | --- | --- |
|  |  |  | M1 | M2 |
| OCLN and PLIN2 colocalization | 0.22±0.16 | 0.99±0.00 | 0.02±0.01 | 0.08±0.10 |
| OCLN and XOR colocalization | 0.40±0.14 | 0.94±0.05 | 0.44±0.20 | 0.40±0.14 |
| OCLN and BTN1A1 colocalization | 0.46±0.17 | 0.96±0.03 | 0.54±0.21 | 0.32±0.16 |

Note based on these coefficients, if n=1, it means two proteins have perfect colocalization; whereas if n=0, it means they do not colocalize.
