# Supplementary material for: Occludin is a target of Src kinase and promotes lipid secretion by binding to BTN1a1 and XOR
Source: PLoS Biol. 2022 Jan 18;20(1):e3001518. doi: 10.1371/journal.pbio.3001518 (PMC8797263; doi:10.1371/journal.pbio.3001518)
Supplement: S2 Table — (DOCX) [file pbio.3001518.s002.docx]

S2 Table. Primers used in qPCR.

| Gene name | Forward sequence (5’ 🡪 3’) | Reverse sequence (5’ 🡪 3’) |
| --- | --- | --- |
| *Lipa* | CCCACCAAGTAGGTGTAGGC | GAGTTGCATCGGGAGTGGTC |
| *Lipg* | TCGGCTTTTGGAGCGTCTAT | TTTATGATGCTCATCTCGCAGC |
| *Lipe* | GGGAGGGCCTCAGCG | AGGCCATATTGTCTTCTGCGA |
| *Gpd1l* | GACCACCATTGGCAGCAAAG | CACGGTGTCTGCATCGTCTA |
| *Gk5* | CTCGCTGTTGGATTTTGGGC | TCTTAAAATGCCTCGTATTCCTGC |
| *Agk* | GCCTGTCAAGAAGCTCAGGT | AGTTCTGGCTTTGCCTTTGC |
| *Acss1* | CGGTTGGATCACAGGACACA | GTCTCCCAGTAACGACCAGC |
| *Acss2* | TCCAGATGTCCAGATCTGCTG | GAGTGGGTCCTCAGCATCAC |
| *Acsl1* | AGCCTCACTGCCCTTTTCT | ATGCAGAATTCATCTGTGCCATC |
| *Dgat1* | CGACGGCTACTGGGATCTGA | CTCAGGATCAGCATCACCACAC |
| *Lpin1* | CCGGCCTGCTGATGTGTATT | GTGATCGACCACTTCGCAGA |
| *Lpin2* | GGACAGCTACGATTACACCATC | GGGAAGCTCAGTAGACGCT |
| *Gpam* | CTTGGCCGATGTAAACACACC | CTTCCGGCTCATAAGGCTCTC |
| *Dgka* | ATGCGAGTGGCCGAATATCTA | CTGCCGTCCTGATCCATCTC |
| *Cidea* | TGACATTCATGGGATTGCAGAC | GGCCAGTTGTGATGACTAAGAC |
| *Plin2 (Adph)* | GTTTTGGGGATGGTGCAGTT | CCAGCCGTTCATAGTTGCTC |
| *Btn1a1* | ACGTCAGAGTCCAAGAAGCAT | AGGCCAGTAAGATGATAGCCA |
| *Xor (Xdh)* | ATGACGAGGACAACGGTAGAT | TCATACTTGGAGATCATCACGGT |
| *Th-pok* | CCCGAGGATGACCTGATTGG | CCTGCGTCCTGATGGTGAG |
| *TDP43* | CCTTTGCAGATGATAAGGTTGCC | TGTGCAGCGTGATGACGAA |
| *Actb* | GGCTGTATTCCCCTCCATCG | CCAGTTGGTAACAATGCCATGT |
| *Gapdh* | TTCACCACCATGGAGAAGGC | CCCTTTTGGCTCCACCCT |
