# Supplementary figures and images for: Occludin is a target of Src kinase and promotes lipid secretion by binding to BTN1a1 and XOR
Source: PLoS Biol. 2022 Jan 18;20(1):e3001518. doi: 10.1371/journal.pbio.3001518 (PMC8797263; doi:10.1371/journal.pbio.3001518)

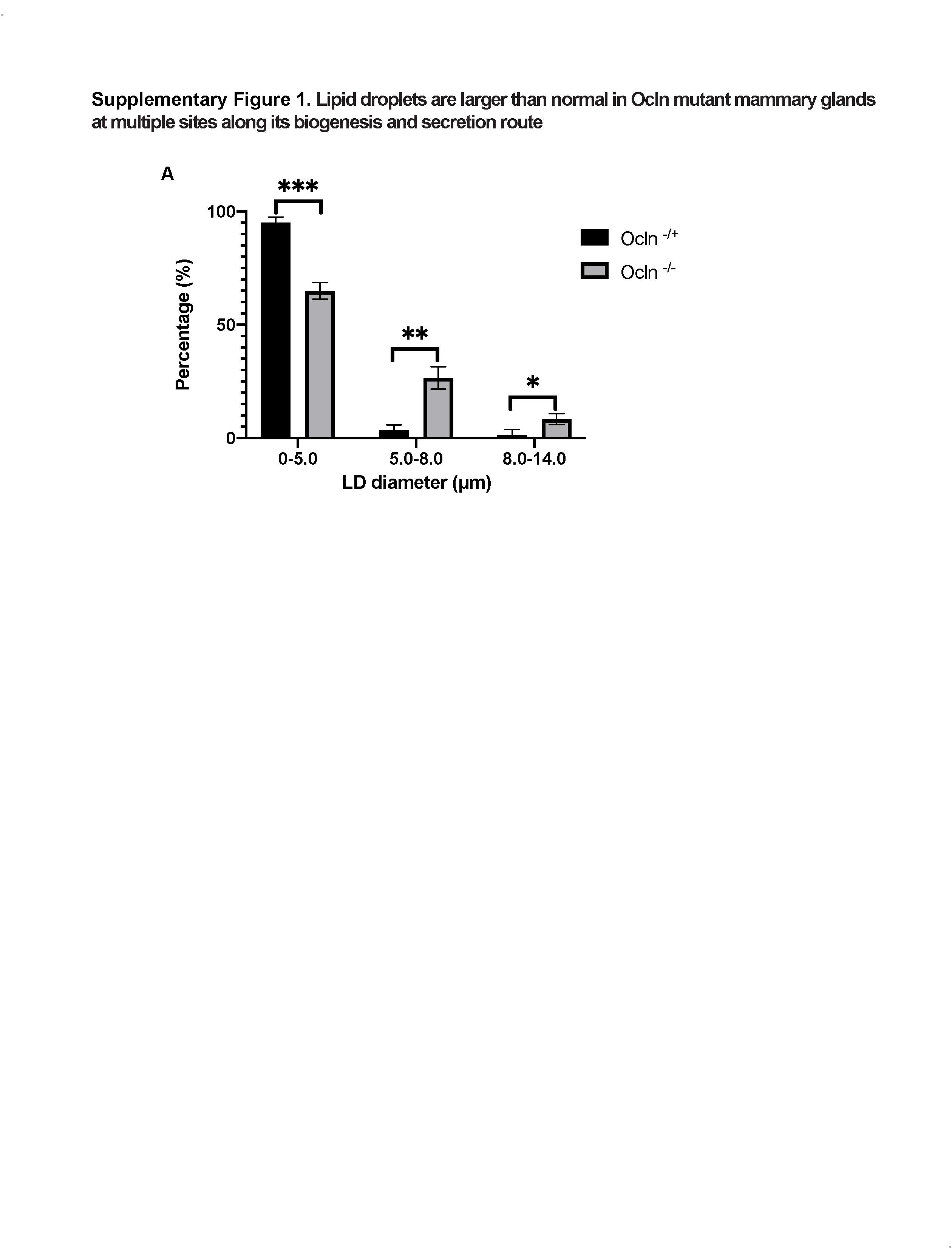

Supplement: S1 Fig — (A) Statistical analysis of LD sizes in Ocln−/+ and Ocln−/− mammary gland alveoli at the L2 stage is shown. t test was used; *P < 0.05, **P < 0.001, ***P < 0.001. LD, lipid droplet; Ocln, Occludin. (TIF) [file pbio.3001518.s003.tif]

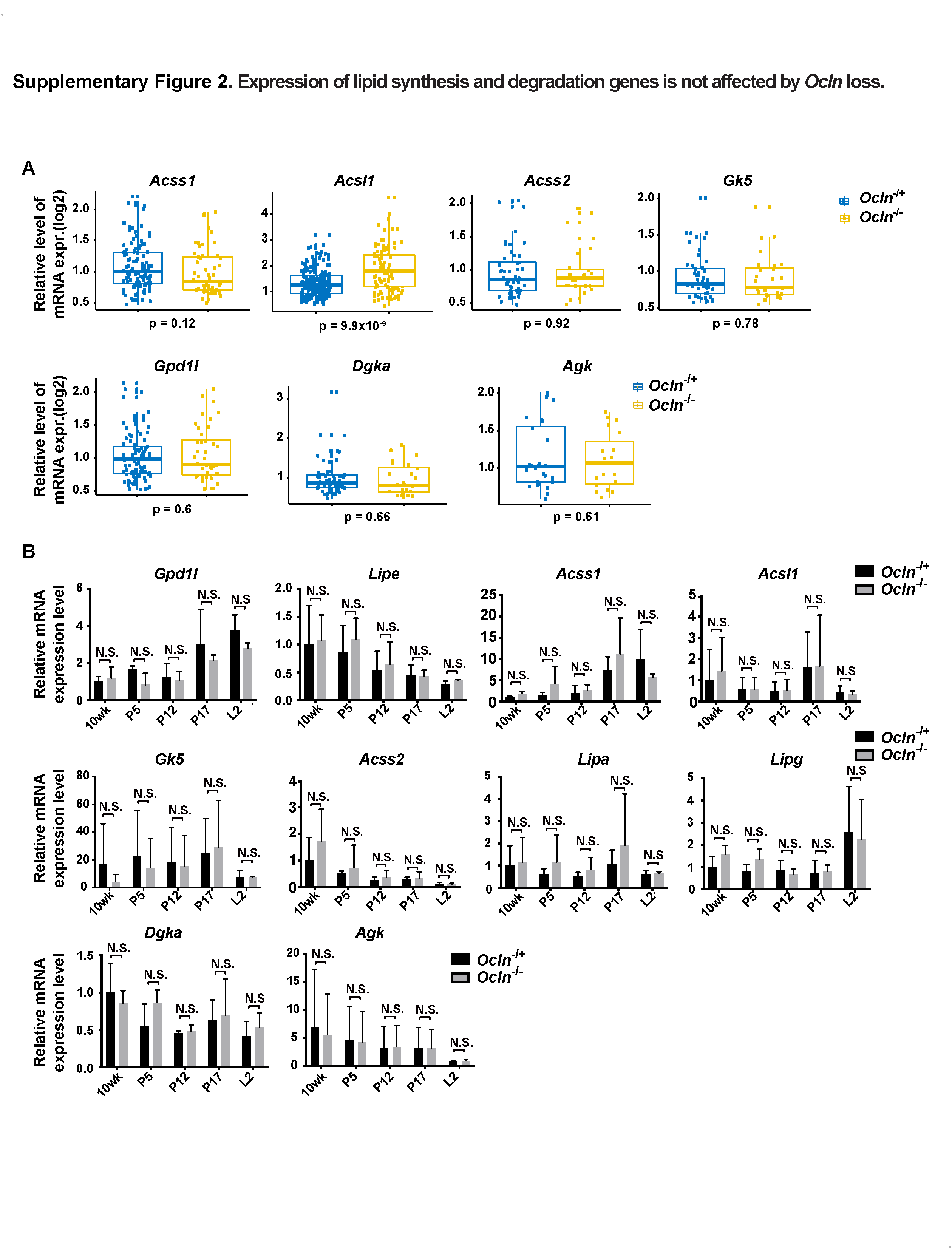

Supplement: S2 Fig — (A) Relative gene expression levels of some of the genes in the TG synthesis pathway are based on data from the scRNA-seq dataset. Each dot indicates the expression level based on log2 of the genes in a single cell. (B) Levels of mRNA expression as detected by qPCR of several key TAG synthesis genes at the 10-wk, P5, P12, P17, and L2 stages. Values were normalized against actin expression, and gene expression at 10 wk of age was set as the base value against which other stages were compared. Graph shows mean ± SD. The number of female mice at each stage used were: Ocln−/+ (n = 3) and Ocln−/− (n = 3). L, lactation; Ocln, Occludin; P, pregnancy; qPCR, quantitative PCR; scRNA-seq, single-cell RNA sequencing; TAG, triacylglycerol; TG, triglyceride. (TIF) [file pbio.3001518.s004.tif]

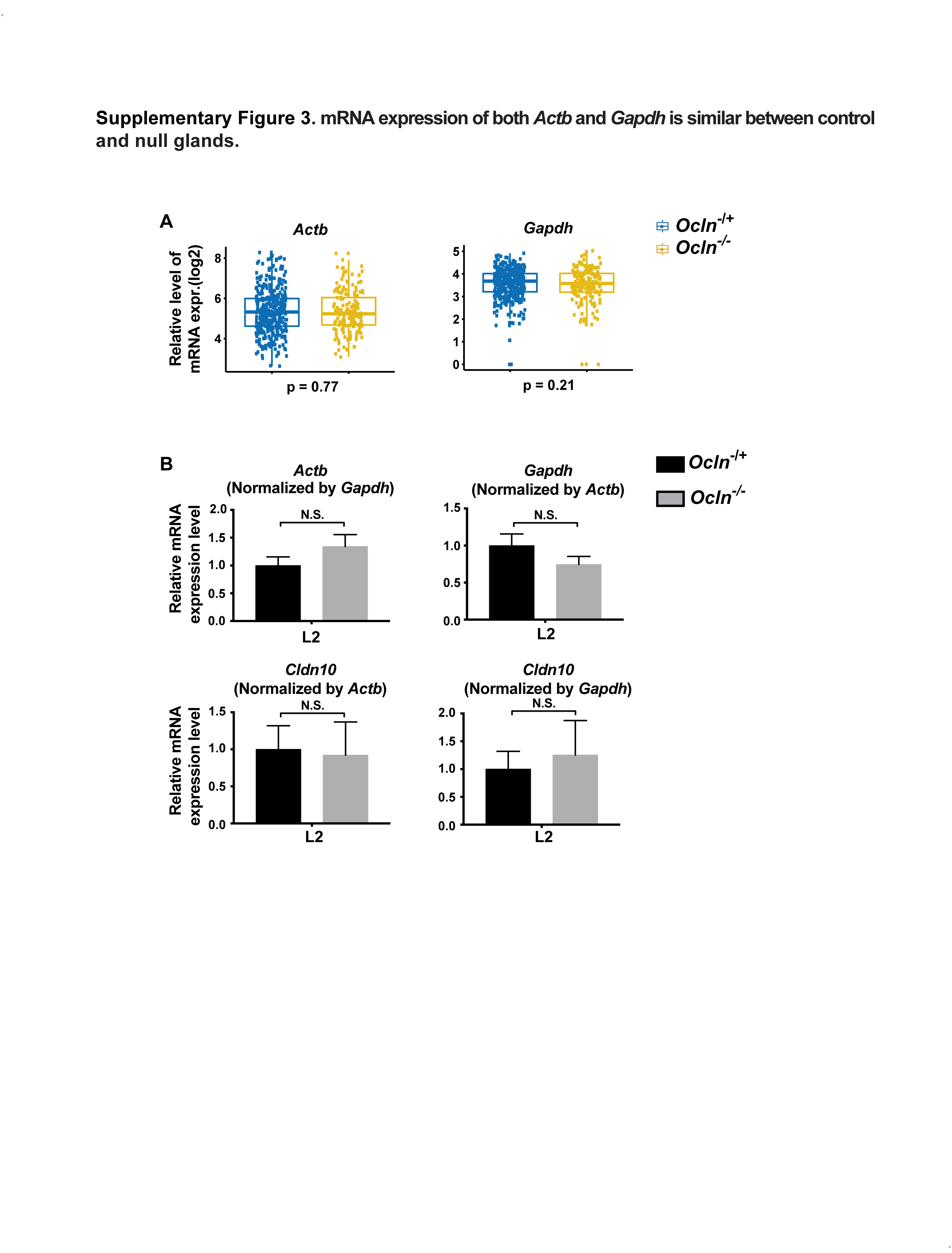

Supplement: S3 Fig — (A) Relative gene expression level of Actb and Gapdh based on data from the scRNA-seq dataset. Each dot indicates the expression level based on log2 of the genes in a single cell. (B) Levels of mRNA expression as detected by qPCR at the L2 stage. Note that expression of Actb, Gapdh, and Cldn10 was comparable between control and Ocln null glands, irrespective of whether Actb or Gapdh was used as an internal control. Graph shows mean ± SD. The number of female mice at the L2 stage used were: Ocln−/+ (n = 3) and Ocln−/− (n = 3). L, lactation; P, pregnancy; qPCR, quantitative PCR; scRNA-seq, single-cell RNA sequencing. (TIF) [file pbio.3001518.s005.tif]

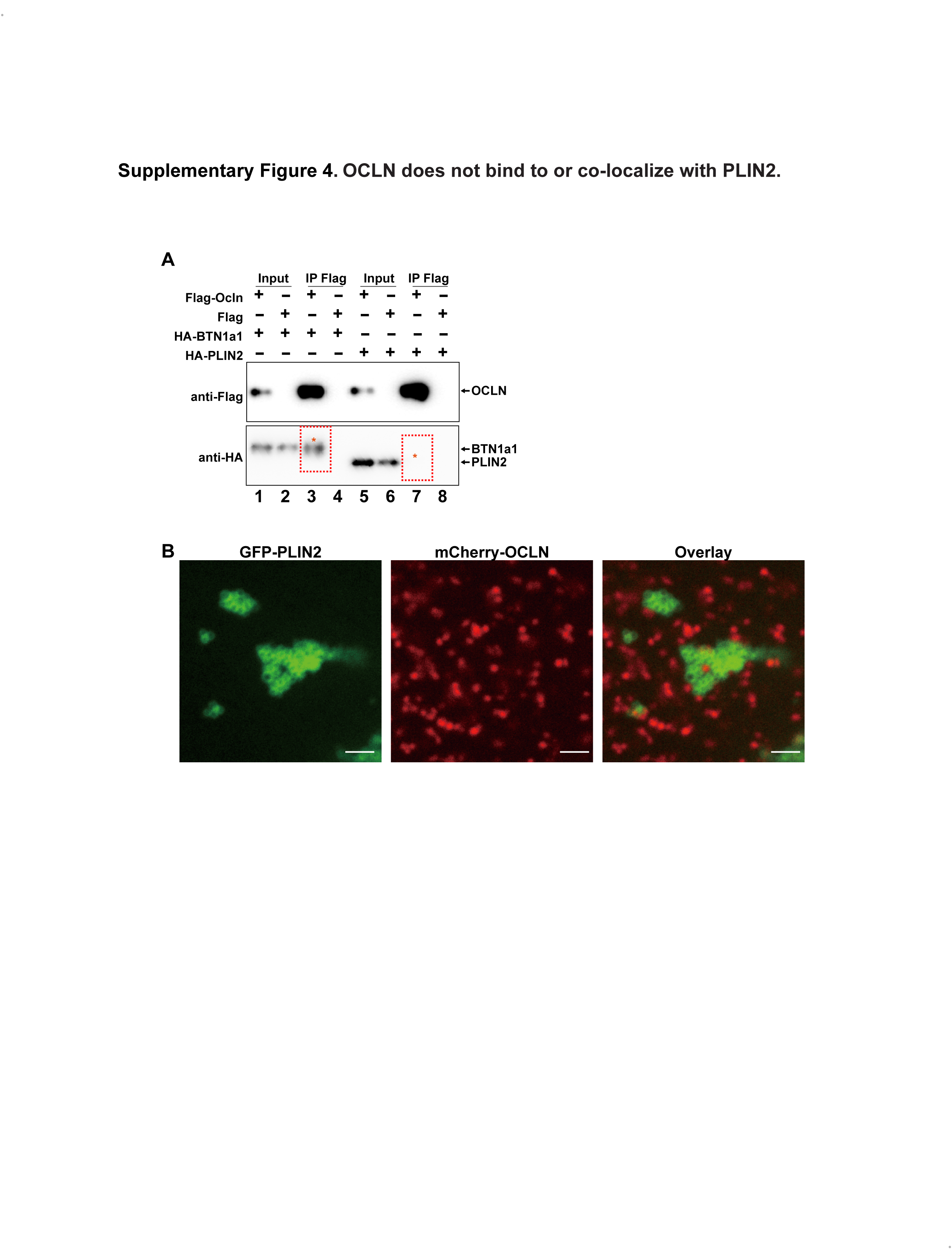

Supplement: S4 Fig — (A) Immunoprecipitation assay to determine protein binding between OCLN and PLIN2. OCLN was tagged by Flag protein, whereas PLIN2 or BTN1a1 was tagged by HA. Antibody against Flag was used for immunoprecipitation, and antibody against HA was used for subsequent western blotting analysis. No binding of PLIN2 to OCLN was detected in this assay (lane 7 marked with an asterisk). The association of BTN1a1 with OCLN was detected in the immunoprecipitation assay (lane 7 marked with an asterisk) and served as a positive control. (B) Time course of localization of OCLN and PLIN2 as detected by fluorescent microscopy. mCherry was fused in-frame with OCLN at the N-terminus, whereas GFP was fused in-frame with PLIN2. Statistical analysis shows that 2% of OCLN particles colocalized with PLIN2 (S1 Table). Scale bars: 2 μm. GFP, green fluorescent protein; HA, hemagglutinin; PLIN2, Perilipin-2. (TIF) [file pbio.3001518.s006.tif]

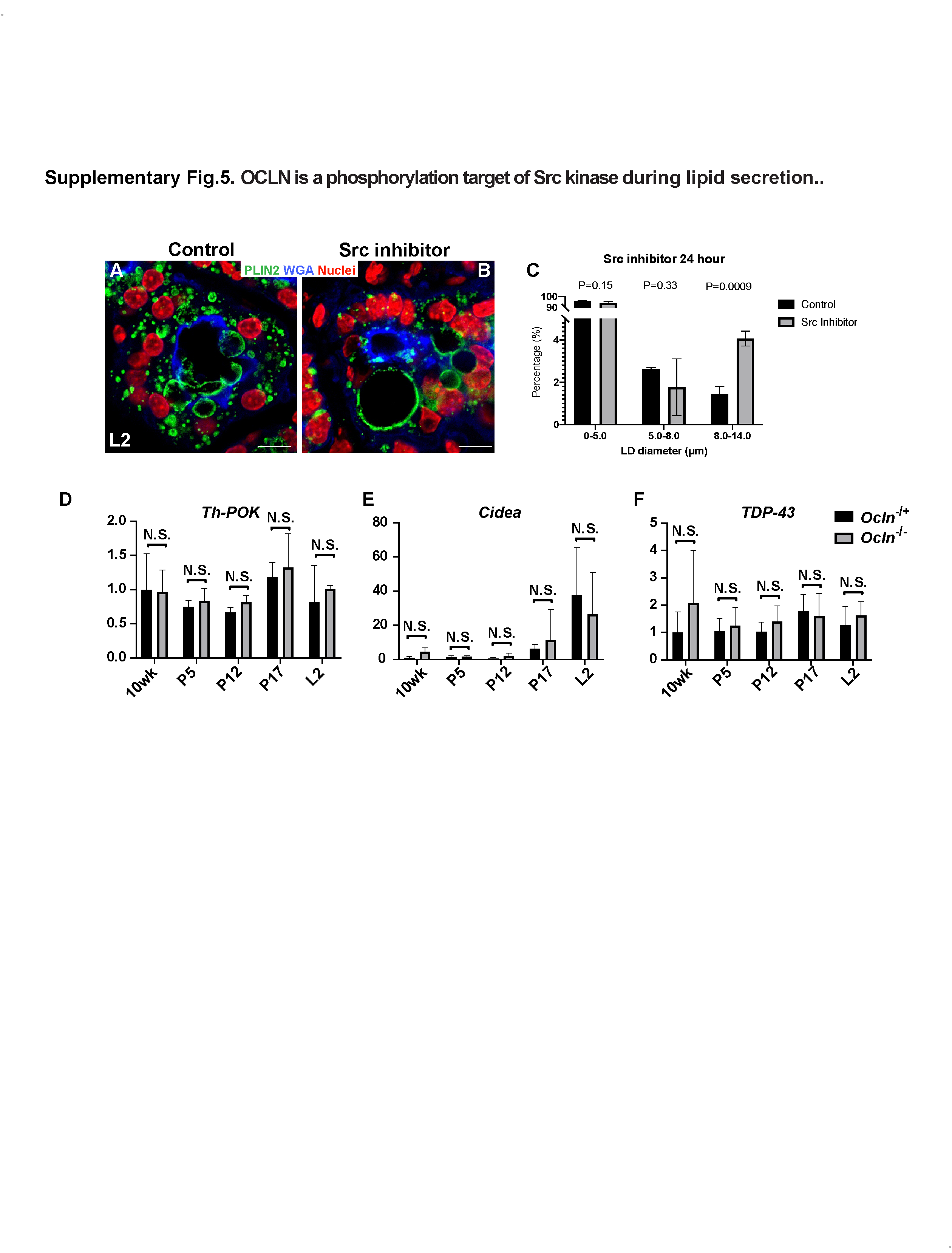

Supplement: S5 Fig — (A-C) Src inhibition impairs LD secretion. LDs as revealed by PLIN2 immunofluorescence (green) on mammary epithelia of control (A) and Src inhibitor-treated (B) mice at the L2 stage. WGA staining (blue) marks the apical surface of the alveoli and is a demarcation of the lumen. Note that LDs have been secreted into the alveolar lumen in the form of MFGs in the control glands. By contrast, while few MFGs are present in the lumen of Src inhibitor-treated glands, a buildup of large LDs are inside epithelial cells of these glands. Samples were counterstained with the nuclear dye DAPI (red). Scale bars: 10 μm. (C) Statistical analysis of LD sizes in control and Src inhibitor-treated mammary gland alveoli at the L2 stage are shown. Note that, while the percentages of small- and medium-sized LDs were indistinguishable between the control and experimental groups, the percentage of large LDs (>4 μm) was significantly more in the Src inhibitor group than in the control group. t test was used. (D-F) Levels of mRNA expression as detected by qPCR of LD secretion regulators, including Th-POK (D), Cidea (E), and TDP-43 (F) in mammary gland epithelial cells at the 10-wk, P5, P12, P17, and L2 stages. Values were normalized against actin expression, and gene expression at 10 wk of age was set as the base value against which other stages were compared. Graph shows mean ± SD. The number of female mice at each stage used were: Ocln−/+ (n = 3) and Ocln−/− (n = 3). L, lactation; LD, lipid droplet; MFG, milk fat globule; P, pregnancy; PLIN2, Perilipin-2; qPCR, quantitative PCR; WGA, wheat germ agglutinin. (TIF) [file pbio.3001518.s007.tif]
