# Supplementary material for: Occludin is a target of Src kinase and promotes lipid secretion by binding to BTN1a1 and XOR
Source: PLoS Biol. 2022 Jan 18;20(1):e3001518. doi: 10.1371/journal.pbio.3001518 (PMC8797263; doi:10.1371/journal.pbio.3001518)

Raw images for the blots in Fig.3E

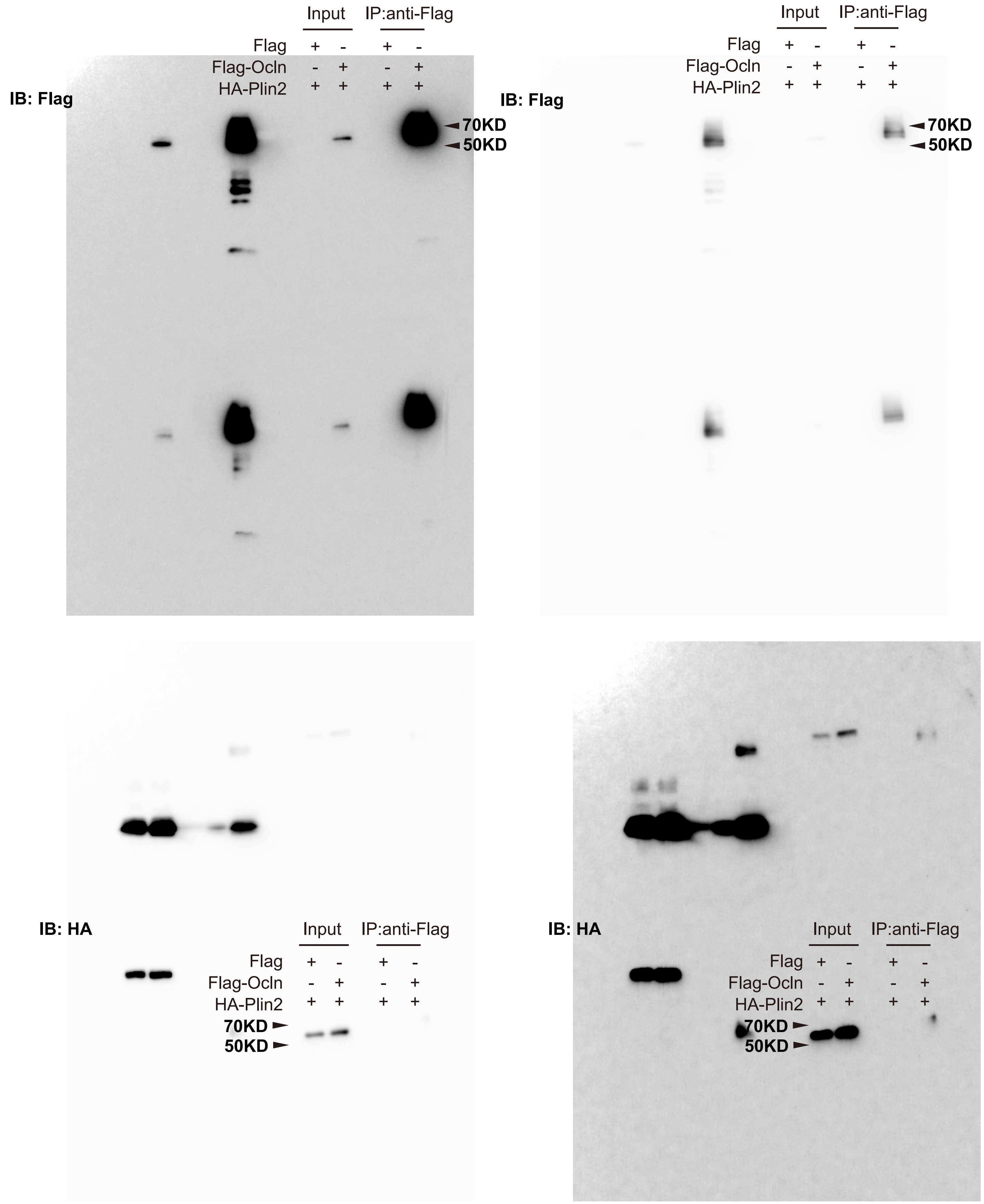

Raw images for the blots in Fig.6C

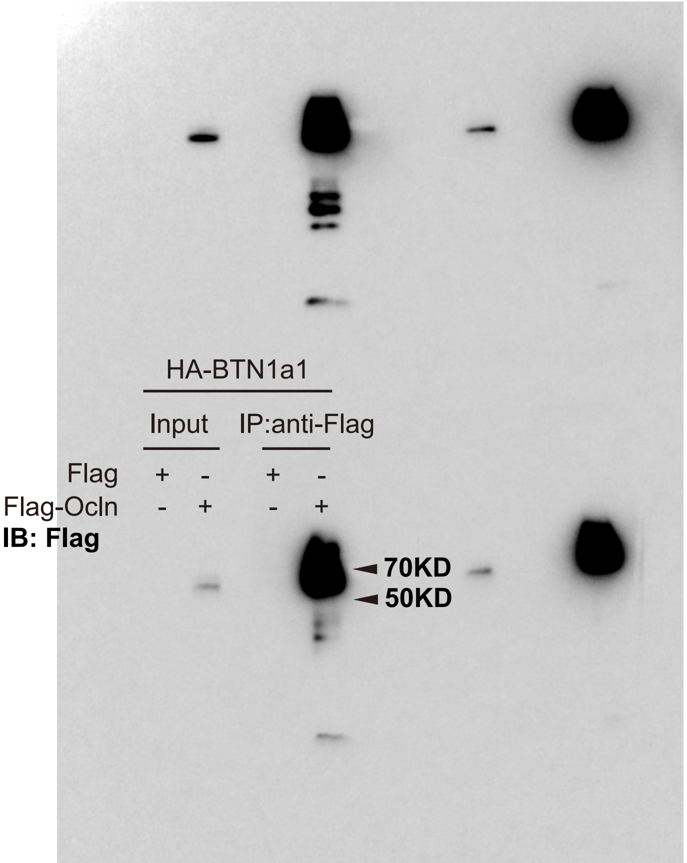

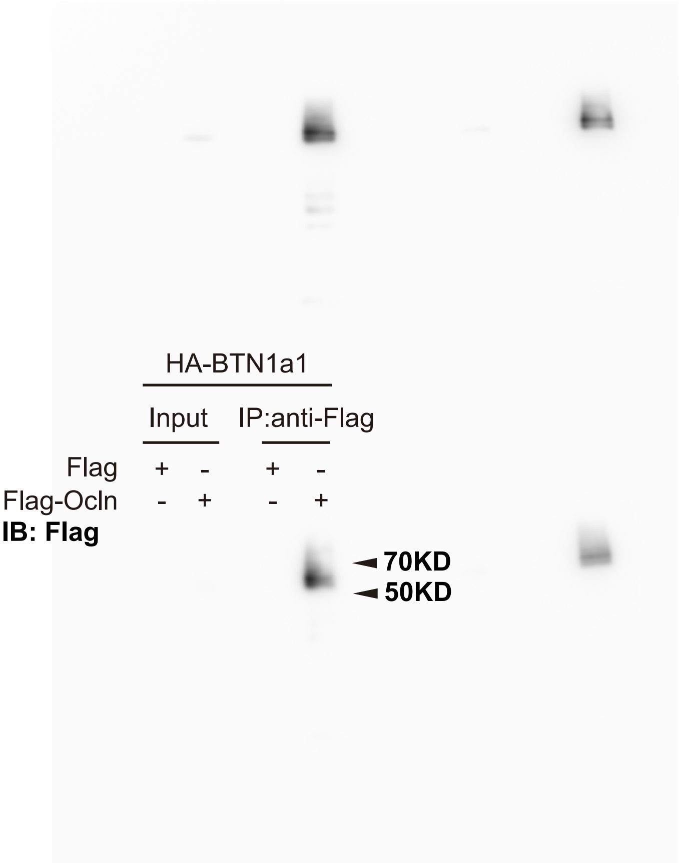

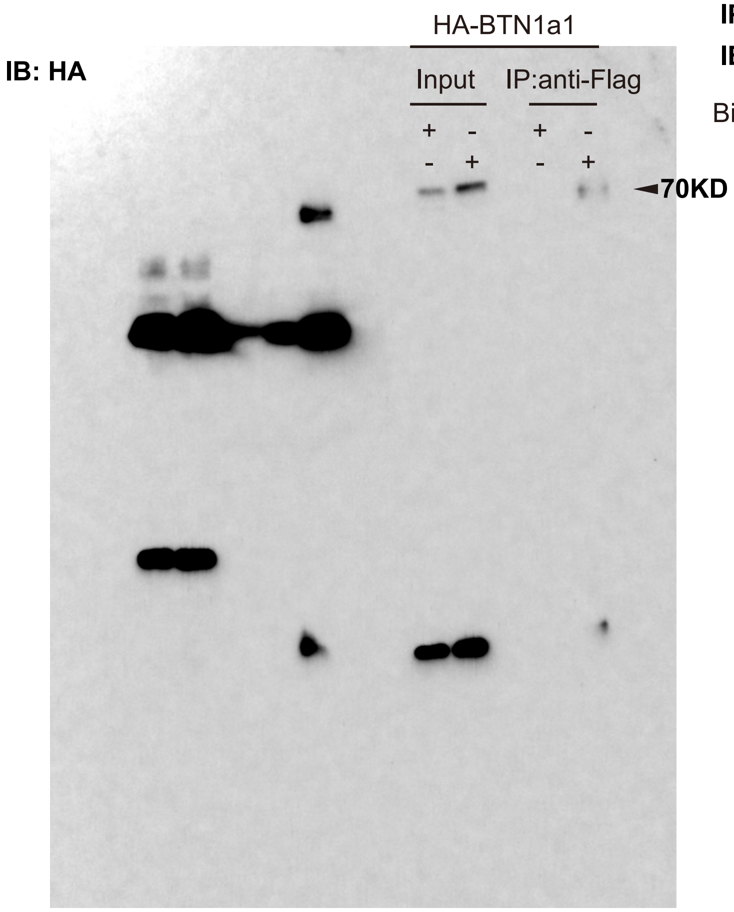

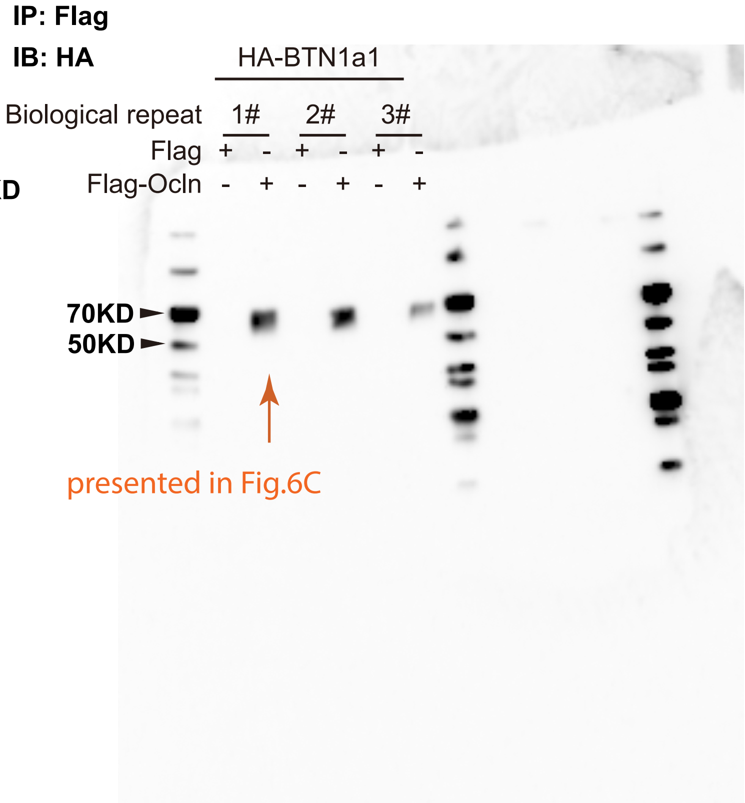

Due to the poor quality of the image,
repeat Western Blot by loading only the IP samples together.

Raw images for the blots in Fig.6D

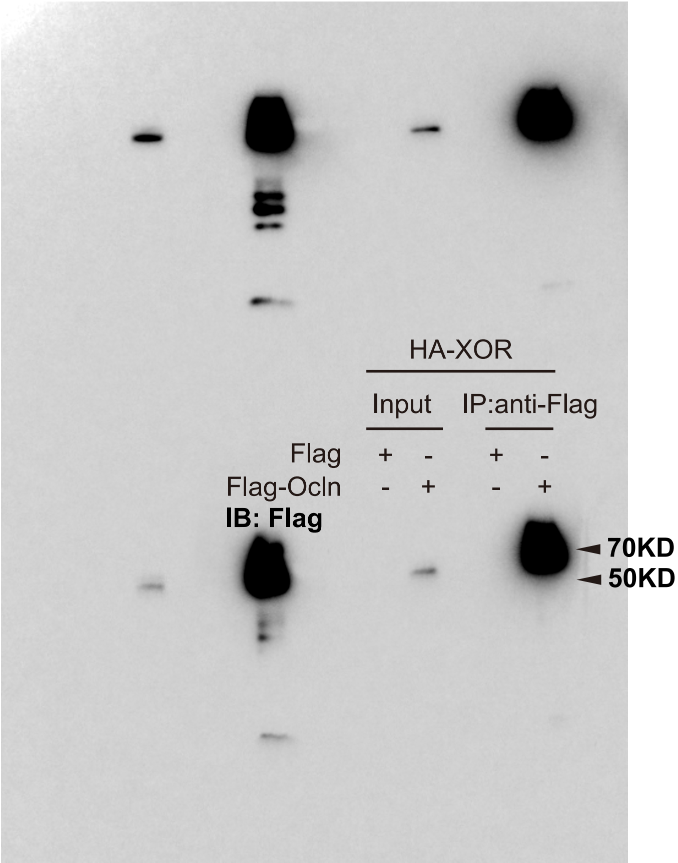

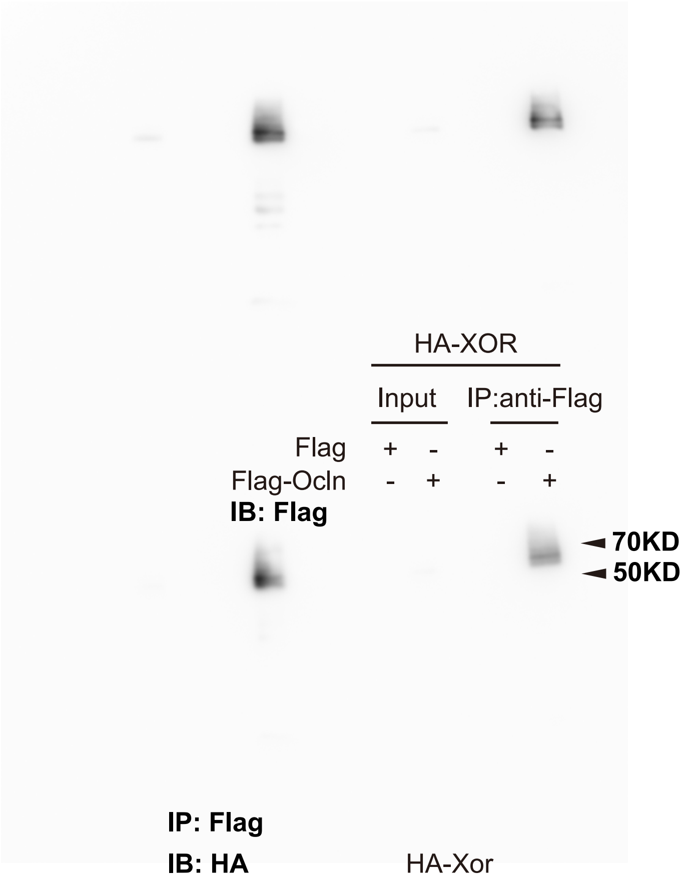

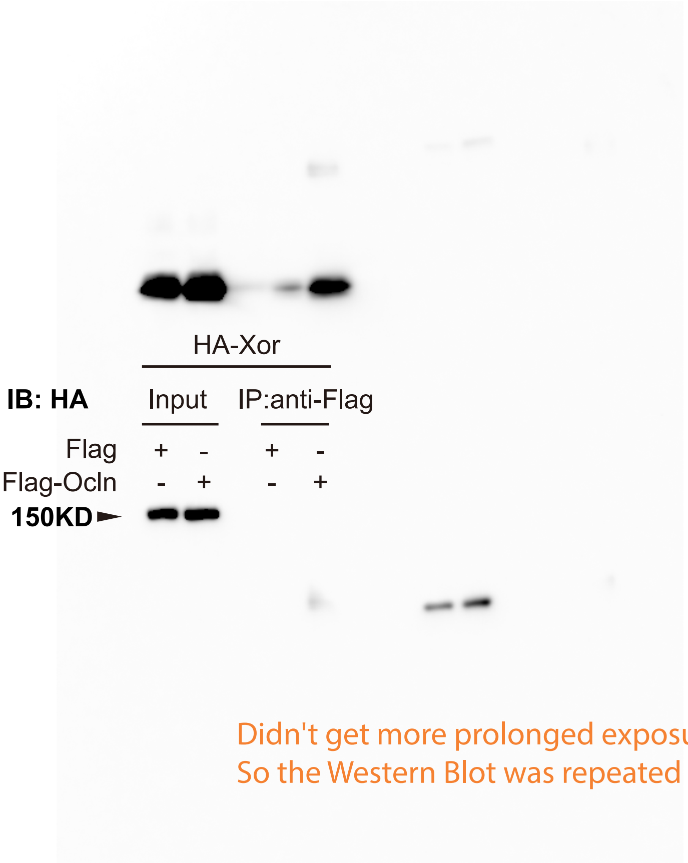

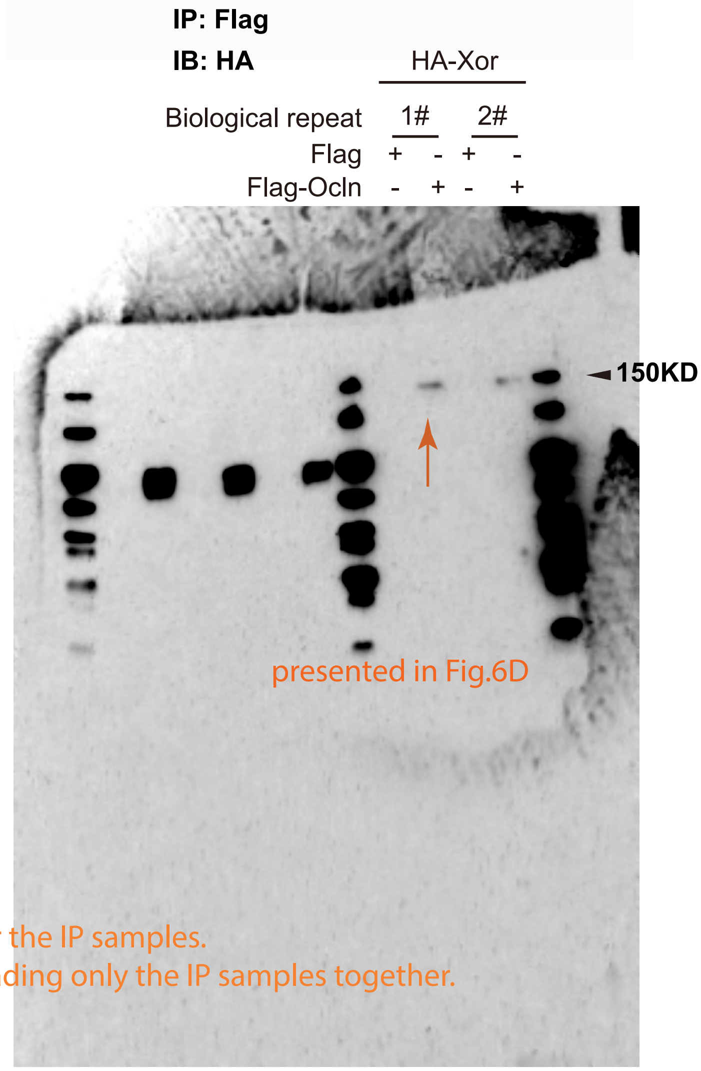

Didn't get more prolonged exposure for the IP samples.
So the Western Blot was repeated by loading only the IP samples together.

Raw images for the blots in Fig.6G/H

IB: HA

	Input				IP:Flag			
FLAG	+	-	-	-	+	-	-	-
FLAG-OCLN	-	+	-	-	-	+	-	-
FLAG-OCLN-ΔN	-	-	+	-	-	-	+	-
FLAG-OCLN-ΔC	-	-	-	+	-	-	-	+
HA-BTN1A1	+	+	+	+	+	+	+	+

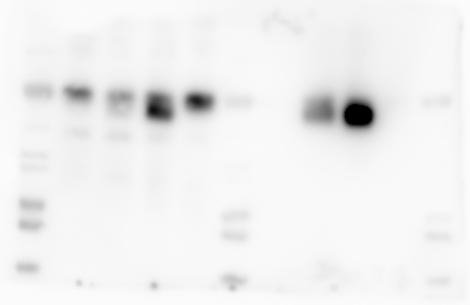

70KD ►

	Input				IP:Flag			
FLAG	+	-	-	-	+	-	-	-
FLAG-OCLN	-	+	-	-	-	+	-	-
FLAG-OCLN-ΔN	-	-	+	-	-	-	+	-
FLAG-OCLN-ΔC	-	-	-	+	-	-	-	+
HA-XOR	+	+	+	+	+	+	+	+

150KD ►

	Input				IP:Flag			
FLAG	+	-	-	-	+	-	-	-
FLAG-OCLN	-	+	-	-	-	+	-	-
FLAG-OCLN-ΔN	-	-	+	-	-	-	+	-
FLAG-OCLN-ΔC	-	-	-	+	-	-	-	+
HA-XOR	+	+	+	+	+	+	+	+

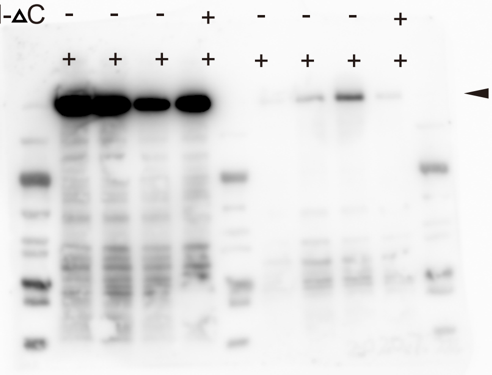

◄ **150KD**

Raw images for the blots in Fig.7A

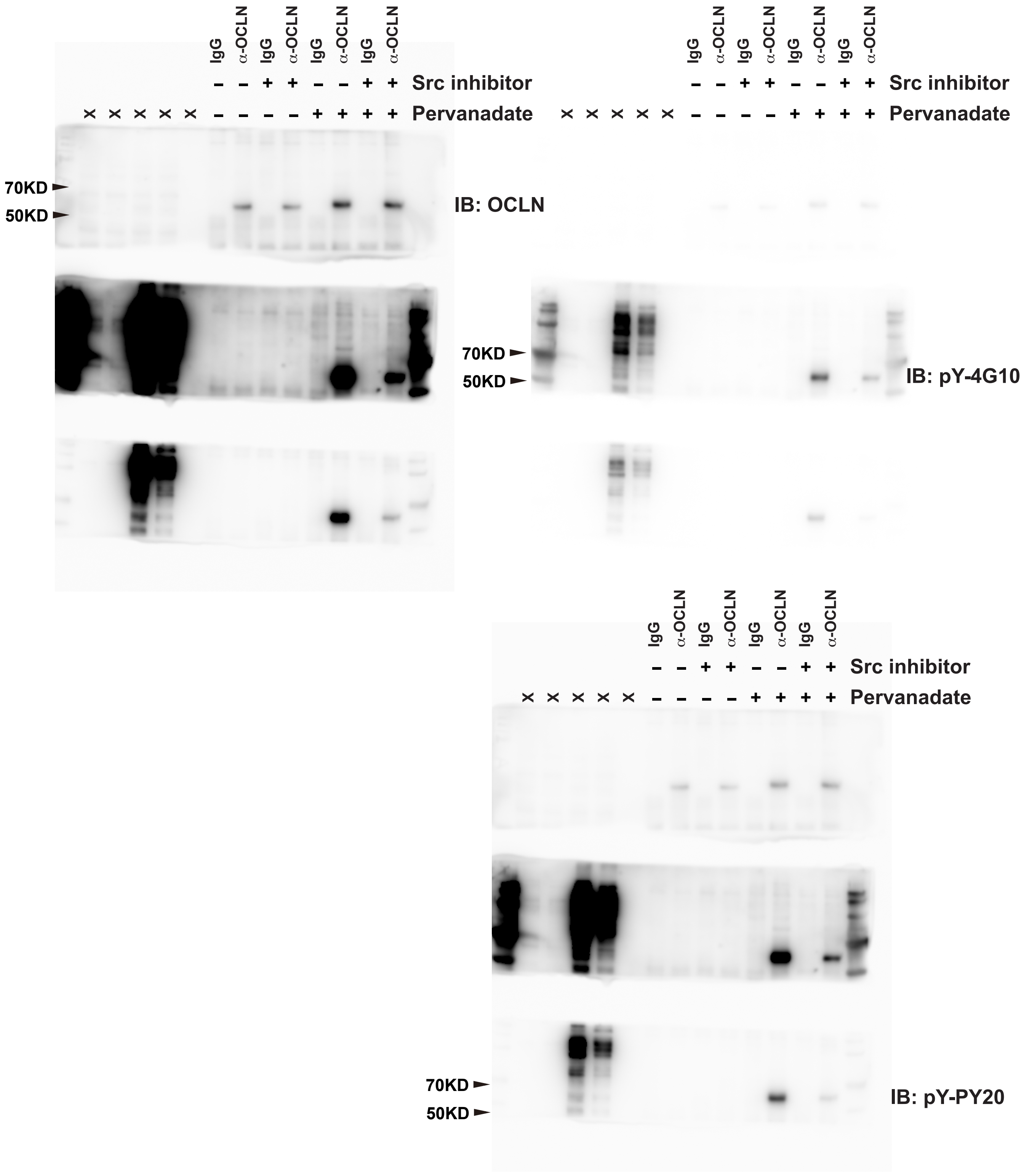

Raw images for the blots in Fig.7D

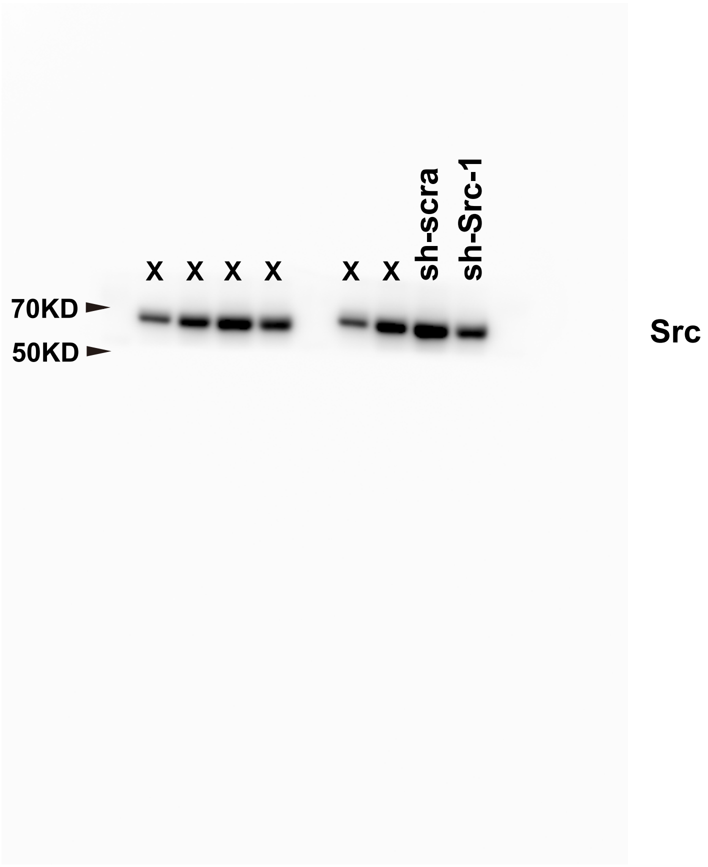

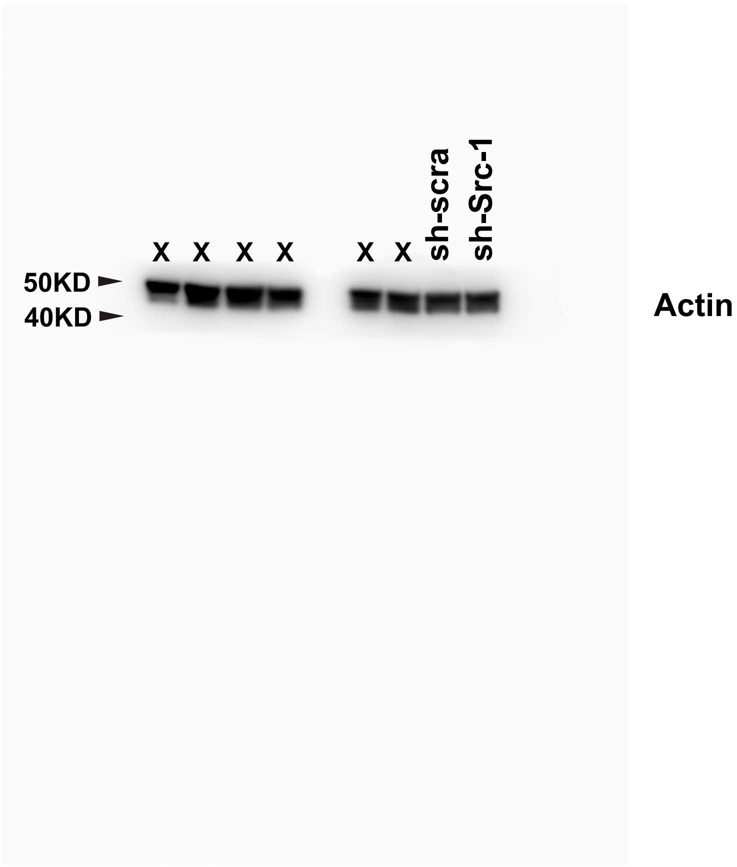

Raw images for the blots in Fig.7F

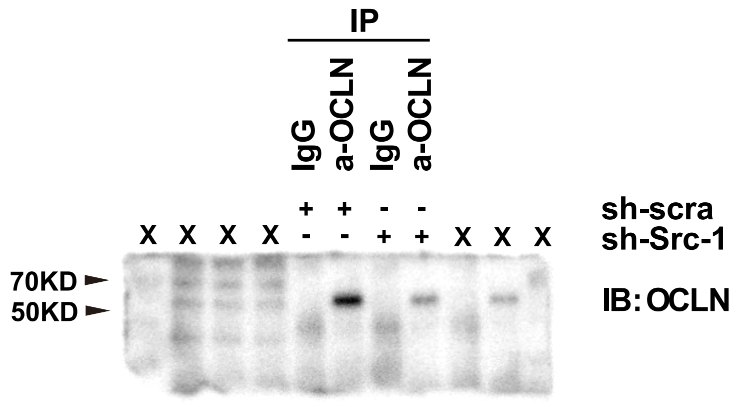

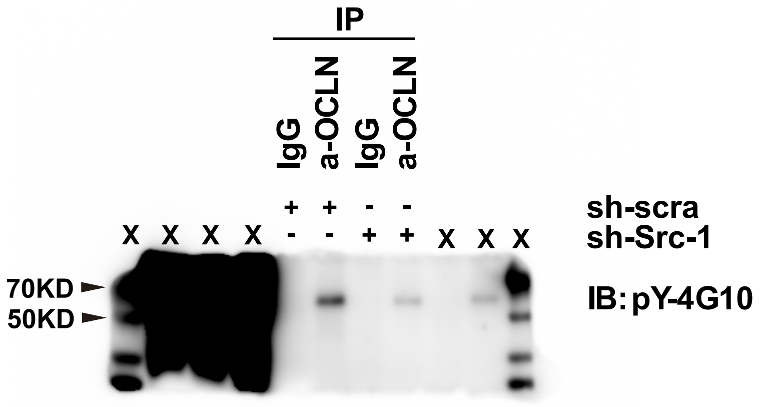

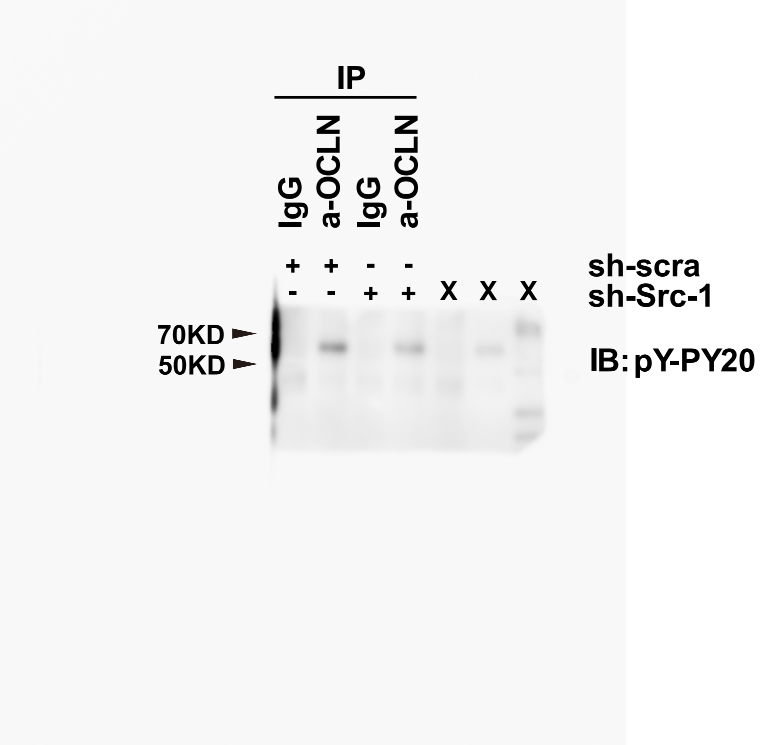

Raw images for the blots in Fig.7H

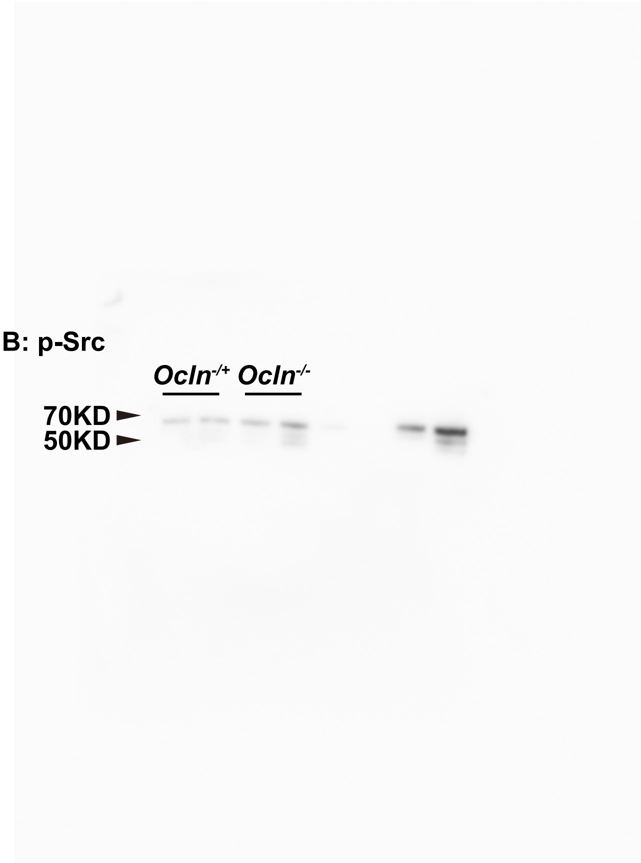

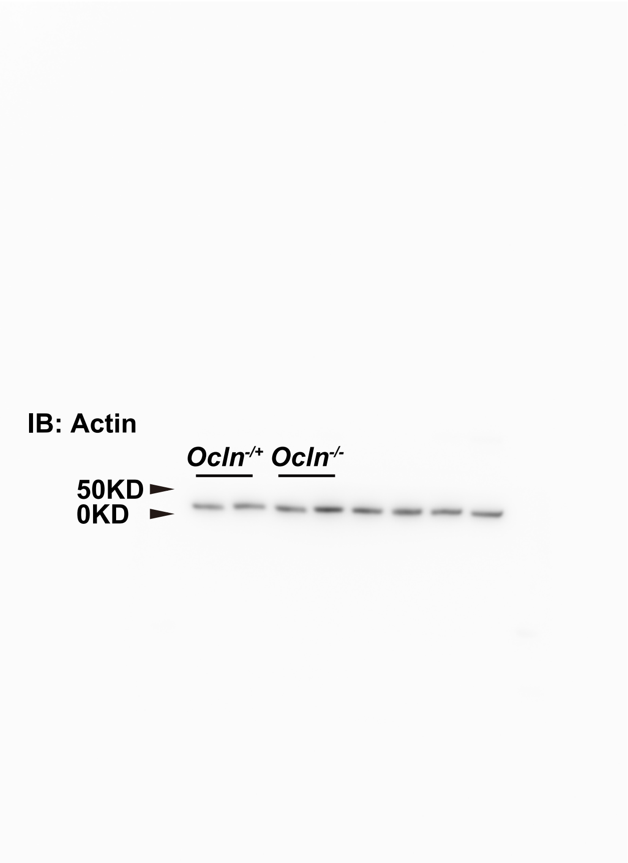

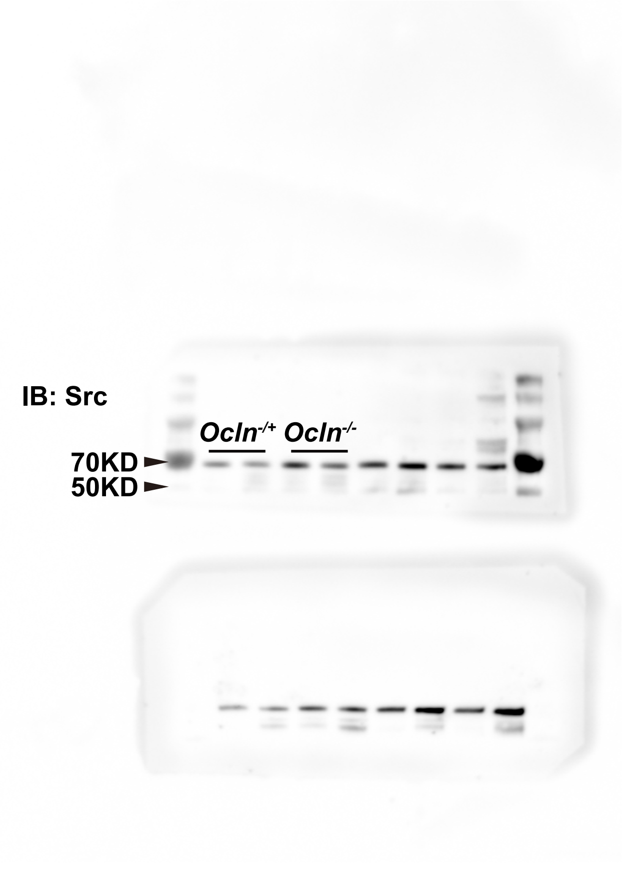

Raw images for the blots in supplementary Fig. 4A

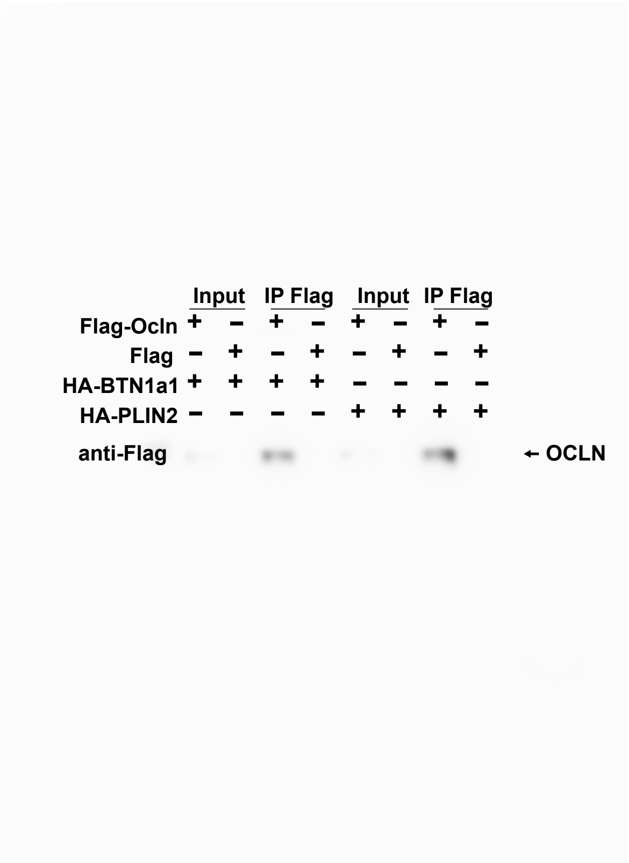

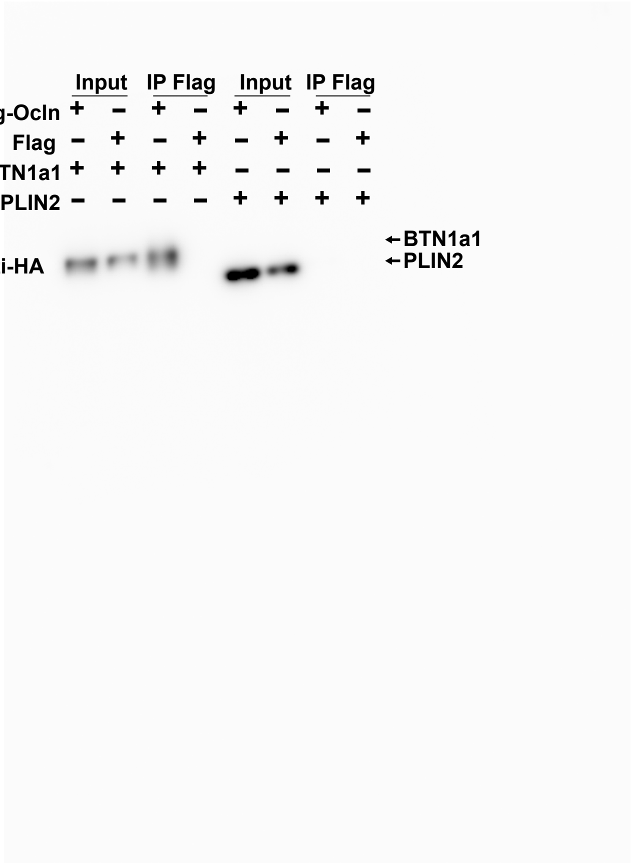

Supplement: S1 Raw image — (PDF) [file pbio.3001518.s012.pdf]
